# Mechanisms of Resistance to Folate Pathway Inhibitors in *Burkholderia pseudomallei*: Deviation from the Norm

**DOI:** 10.1128/mBio.01357-17

**Published:** 2017-09-05

**Authors:** Nicole L. Podnecky, Katherine A. Rhodes, Takehiko Mima, Heather R. Drew, Sunisa Chirakul, Vanaporn Wuthiekanun, James M. Schupp, Derek S. Sarovich, Bart J. Currie, Paul Keim, Herbert P. Schweizer

**Affiliations:** a Department of Microbiology, Immunology and Pathology, College of Veterinary Medicine and Biomedical Sciences, Colorado State University, Fort Collins, Colorado, USA; b Department of Molecular Genetics and Microbiology, College of Medicine, Emerging Pathogens Institute, Institute for Therapeutic Innovation, University of Florida, Gainesville, Florida, USA; c Mahidol-Oxford Tropical Medicine Research Unit, Faculty of Tropical Medicine, Mahidol University, Bangkok, Thailand; d Translational Genomics Research Institute, Flagstaff, Arizona, USA; e Global and Tropical Health Division, Menzies School of Health Research, Darwin, Northern Territory, Australia; f Centre for Animal Health Innovation, University of the Sunshine Coast, Queensland, Australia; g The Pathogen and Microbiome Institute, Northern Arizona University, Flagstaff, Arizona, USA; University of British Columbia

**Keywords:** *Burkholderia*, antibiotic, drug resistance mechanisms, efflux pumps, melioidosis

## Abstract

The trimethoprim and sulfamethoxazole combination, co-trimoxazole, plays a vital role in the treatment of *Burkholderia pseudomallei* infections. Previous studies demonstrated that the *B. pseudomallei* BpeEF-OprC efflux pump confers widespread trimethoprim resistance in clinical and environmental isolates, but this is not accompanied by significant resistance to co-trimoxazole. Using the excluded select-agent strain *B. pseudomallei* Bp82, we now show that *in vitro* acquired trimethoprim versus co-trimoxazole resistance is mainly mediated by constitutive BpeEF-OprC expression due to *bpeT* mutations or by BpeEF-OprC overexpression due to *bpeS* mutations. Mutations in *bpeT* affect the carboxy-terminal effector-binding domain of the BpeT LysR-type activator protein. Trimethoprim resistance can also be mediated by dihydrofolate reductase (FolA) target mutations, but this occurs rarely unless BpeEF-OprC is absent. BpeS is a transcriptional regulator that is 62% identical to BpeT. Mutations affecting the BpeS DNA-binding or carboxy-terminal effector-binding domains result in constitutive BpeEF-OprC overexpression, leading to trimethoprim and sulfamethoxazole efflux and thus to co-trimoxazole resistance. The majority of laboratory-selected co-trimoxazole-resistant mutants often also contain mutations in *folM*, encoding a pterin reductase. Genetic analyses of these mutants established that both *bpeS* mutations and *folM* mutations contribute to co-trimoxazole resistance, although the exact role of *folM* remains to be determined. Mutations affecting *bpeT*, *bpeS*, and *folM* are common in co-trimoxazole-resistant clinical isolates, indicating that mutations affecting these genes are clinically significant. Co-trimoxazole resistance in *B. pseudomallei* is a complex phenomenon, which may explain why resistance to this drug is rare in this bacterium.

## IMPORTANCE

*Burkholderia pseudomallei* causes melioidosis, a tropical disease that is difficult to treat. The bacterium’s resistance to antibiotics limits therapeutic options. The paucity of orally available drugs further complicates therapy. The oral drug of choice is co-trimoxazole, a combination of trimethoprim and sulfamethoxazole. These antibiotics target two distinct enzymes, FolA (dihydrofolate reductase) and FolP (dihydropteroate synthase), in the bacterial tetrahydrofolate biosynthetic pathway. Although co-trimoxazole resistance is minimized due to two-target inhibition, bacterial resistance due to *folA* and *folP* mutations does occur. Co-trimoxazole resistance in *B. pseudomallei* is rare and has not yet been studied. Co-trimoxazole resistance in this bacterium employs a novel strategy involving differential regulation of BpeEF-OprC efflux pump expression that determines the drug resistance profile. Contributing are mutations affecting *folA*, but not *folP*, and *folM*, a folate pathway-associated gene whose function is not yet well understood and which has not been previously implicated in folate inhibitor resistance in clinical isolates.

## INTRODUCTION

While *Burkholderia pseudomallei* and melioidosis, the disease that it causes, were traditionally documented mainly in northern Australia and Southeast Asia, it has now been established that the bacterium is endemic to many parts of the tropics, including the Americas, the Indian subcontinent, other parts of Asia, and Africa (1–7). Melioidosis is a multifaceted disease that is difficult to treat (2, 8, 9), mostly due to a cadre of chromosomally encoded drug resistance mechanisms similar to those found in other Gram-negative bacteria (10). Unlike many other Gram-negative bacteria, however, the acquisition of horizontally transferred resistance mechanisms has not yet been documented in *B. pseudomallei*.

**FIG 1:**
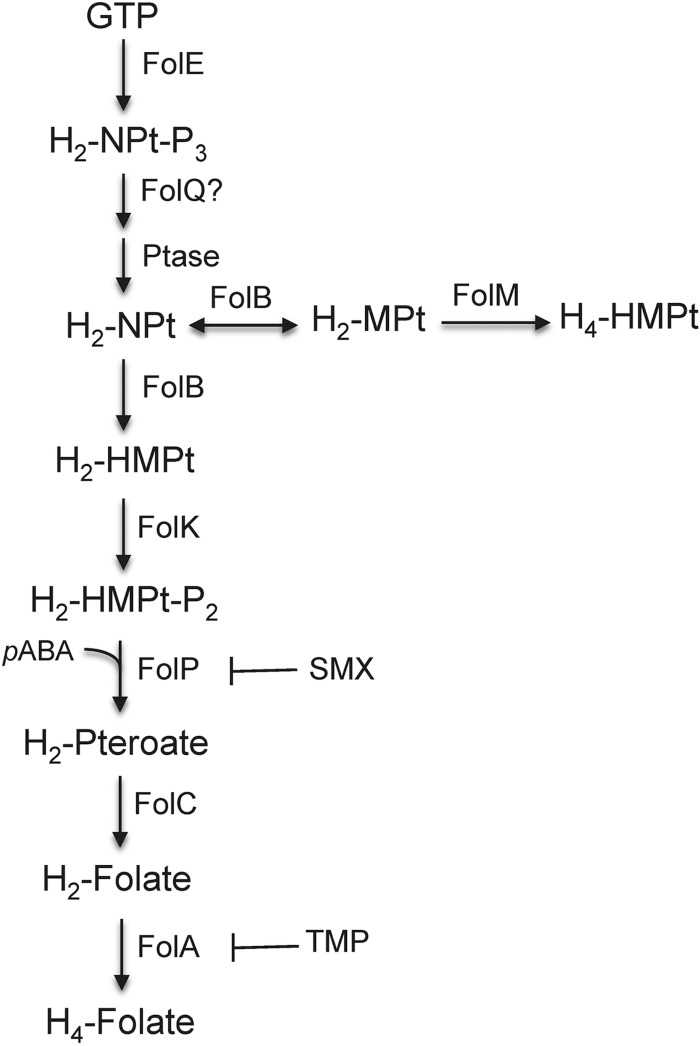
Proposed *B. pseudomallei* tetrahydrofolate and tetrahydromonopterate synthesis pathways. The pathways are based on tetrahydrofolate (H_4_-Folate) and tetrahydromonopterate (H_4_-MPt) biosynthetic pathways that have been either established (H_4_-Folate) or proposed (H_4_-MPt) (14) in other bacteria. Genes for all indicated enzymes are present in annotated *B. pseudomallei* genomes. The *folQ* question mark indicates that the *Burkholderia* homolog is not known. The enzymatic steps of the H_4_-Folate pathway that are inhibited by either sulfamethoxazole (SMX) or trimethoprim (TMP) are indicated. Abbreviated enzyme names: FolA, dihydrofolate reductase; FolB, dihydroneopterin aldolase/epimerase; FolC, dihydrofolate synthetase; FolE, GTP cyclohydrolase I; FolK, hydroxymethyl-dihydropterin pyrophosphokinase; FolM, dihydromonapterin reductase; FolP, dihydropteroate synthase; FolQ, dihydroneopterin triphosphate pyrophosphohydrolase; Ptase, nonspecific phosphohydrolase. Full metabolite names: H_2_-NPt-P_3_, 7,8-dihydroneopterin triphosphate; H_2_-NPt, 7,8-dihydroneopterin; H_2_-HMPt, 6-hydroxymethyl-7,8-dihydropterin; H_2_-HMPt-P_2_, 6-hydroxymethyl-7,8-dihydropterin diphosphate; H_2_-Pteroate, 7,8-dihydropteroate; H_2_-Folate, 7,8-dihydrofolate; H_4_-Folate, tetrahydrofolate; H_2_-MPt-P_3_, 7,8-dihydromonapterin triphosphate; H_2_-MPt, 7,8-dihydromonapterin; H_4_-MPt, tetrahydromonapterin; *p*-ABA, *p*-aminobenzoic acid.

**FIG 2:**
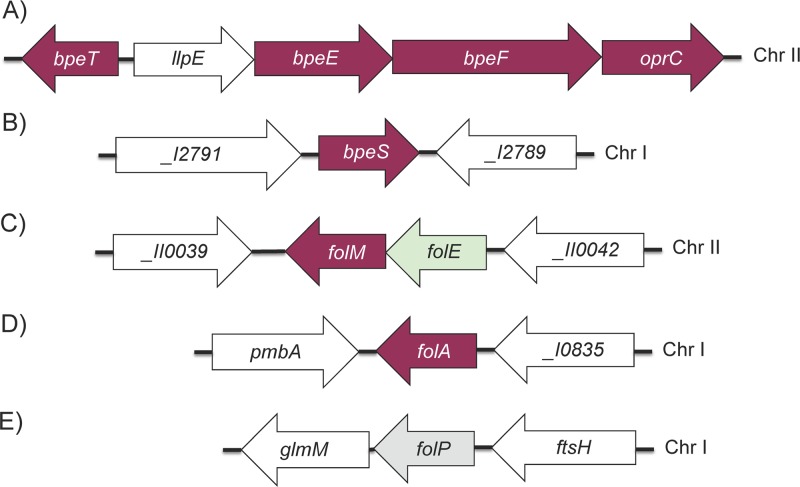
Genomic organization of *B. pseudomallei* genes involved in trimethoprim and sulfamethoxazole resistance. Genes shown in our studies to be involved in *B. pseudomallei* TMP, SMX, and SXT resistance are indicated in maroon. The *folP* gene (indicated in gray) was examined for mutations in this study but was not directly implicated in folate pathway inhibitor resistance. The *folE* gene is indicated in light green. Sequence coordinates are taken from the GenBank entries for strain 1026b (GenBank accession numbers NC_017831.1 and NC_017832.1) and from the *Burkholderia* database (18). Old NCBI locus tags are used throughout this paper as they are employed in the *Burkholderia* database. Where available, current NCBI locus tags for selected genes are provided below. (A) Organization of the *llpE-bpeE-bpeF-oprC* operon and associated *bpeT* gene on chromosome II (Chr II). The *bpeE*, *bpeF*, and *oprC* genes encode the membrane fusion protein, RND transporter, and outer membrane channel protein components of the BpeEF-OprC efflux pump, respectively. We confirmed that *llpE* is cotranscribed with the *bpeEF-oprC* genes in the *llpE*-*bpeE-bpeF-oprC* operon; however, its product LlpE (putative lipase/carboxyl esterase) does not affect efflux pump function, at least not export of known antibiotic substrates (T. Mima and H. P. Schweizer, unpublished data). The *bpeT* gene encodes a LysR-type regulator, which binds to the 188-bp *bpeT*-*llpE* intergenic region. (B) Chromosome I location of the *bpeS* gene encoding the LysR-type regulator BpeS, which is highly similar to BpeT. The *bpeS* flanking genes are annotated _*I2791* (*BP1026B*_*I2791*; current NCBI tag BP1026B_RS13955, peptidase) and _*I2789* (*BP1026B*_*I2789*; current NCBI tag BP1026B_RS13950, two-component regulator histidine sensor kinase). (C) Location of *folM* and *folE* on chromosome II. The *folM* gene (dihydromonapterin reductase) is in an operon with *folE*, encoding GTP cyclohydrolase (I) catalyzing the first step in the canonical tetrahydrofolate biosynthetic pathway (Fig. 1) (54). We have shown that *B. pseudomallei folE* can complement an *E. coli folE* mutant (Fig. S4). Neighboring genes are _*II0039* (*BP1026B*_*II0039*; current NCBI tag BP1026B_RS18775, serine *O*-acetyltransferase) and _*II0042* (*BP1026B*_*II0042*; current NCBI tag BP1026B_RS18770, LysR-type transcriptional regulator). (D) Location of the dihydrofolate reductase gene *folA* on chromosome I. The *folA* gene is flanked by *pmbA*, encoding a protein belonging to the peptidase U62 family, and _*I0835* (BP1026B_I0835), encoding a putative σ_54_-dependent transcriptional regulator. In some isolates, e.g., the originally sequenced strain K96243 (55), a copy of insertion element IS*Bma2* is located between *pmbA* and *folA* (28). (E) Location of *folP* on chromosome I. The *folP* gene (dihydropteroate synthase) is located upstream of and probably in the same operon as *glmM* (phosphoglucosamine mutase). The preceding gene, *ftsH*, encodes a cell division protein.

Standard therapy for *B. pseudomallei* infection is divided into two phases: the acute phase and the eradication phase. The trimethoprim (TMP)-sulfamethoxazole (SMX) combination co-trimoxazole (SXT) is considered the standard for melioidosis eradication-phase therapy and is also the drug of choice for postexposure therapy (9, 11). TMP and SMX inhibit separate enzymes in the *de novo* bacterial tetrahydrofolic acid synthesis pathway, namely, dihydrofolate reductase (DHFR), encoded by *folA*, and dihydropteroate synthetase (DHPS), encoded by *folP* (Fig. 1) (12). Many bacteria possess *folM*, encoding a still somewhat mysterious enzyme, which in *Escherichia coli* exhibits weak DHFR activity *in vitro* (13). This, however, is likely not its biological function because DHFR activity is normally mediated by FolA, which is an essential enzyme, and dihydrofolate is not a good substrate for FolM (14). FolM is very similar to *Leishmania* Ptr1, which is a NADPH-dependent reductase that reduces various dihydropterins to the tetrahydro state (15). *Leishmania* and related trypanosomatid protozoans lack a *de novo* pteridine biosynthetic pathway and are thought to rely on salvage of both pterins and folates (16). In bacteria such as *Burkholderia* spp. that possess a *de novo* pteridine biosynthetic pathway and phenylalanine hydroxylase (PhhA), FolM likely provides the essential tetrahydromonapterine cofactor for PhhA (14). In these bacteria, *folM* often clusters with *folE*, which encodes a GTP hydrolase that catalyzes the first step of the *de novo* tetrahydofolate biosynthetic pathway found in most bacteria (Fig. 1) (17). *B. pseudomallei* chromosome 2 encodes a gene (*BP1026B_II0040*) that is annotated as *ptr1*, forms an operon with *folE* (Fig. 2) (18), and is likely a FolM homolog.

10.1128/mBio.01357-17.5 FIG S4  *Burkholderia pseudomallei folE* encodes GTP cyclohydrolase I. Download FIG S4, PDF file, 0.1 MB. Copyright © 2017 Podnecky et al. 2017 Podnecky et al. This content is distributed under the terms of the Creative Commons Attribution 4.0 International license.

DHFR mutations are frequently found in TMP-resistant (TMP^r^) bacteria (12, 19–21). Mutations affecting DHPS confer SMX^r^ in a range of Gram-positive and Gram-negative bacteria (12, 20, 22). The true prevalence of SXT resistance (SXT^r^) in *B. pseudomallei* has been controversial, but recent studies indicate that primary resistance to this drug is uncommon (<1%) (23, 24). To our knowledge, there have been no reports functionally characterizing SMX or SXT resistance mechanisms in *B. pseudomallei*.

We have previously shown by heterologous expression in *Pseudomonas aeruginosa* that *B. pseudomallei* BpeEF-OprC, an efflux pump of the resistance nodulation cell division (RND) family, extrudes chloramphenicol and TMP (25). BpeEF-OprC is a homolog of *B. cenocepacia* CeoAB-OpcM (26) and *P. aeruginosa* MexEF-OprN (27), and both of these pumps can efflux TMP. Furthermore, BpeEF-OprC is responsible for the widespread TMP^r^ in *B. pseudomallei* isolates, although this resistance is not accompanied by significant resistance to SXT (28). Efflux is likely to play a significant role in SXT^r^, which is supported by the observation that laboratory-selected chloramphenicol-resistant *B. thailandensis* mutants expressing BpeEF-OprC are resistant to TMP and SXT (29). Unfortunately, the molecular determinants governing this resistance were not established.

The mechanisms that control BpeEF-OprC expression have yet to be fully characterized. In *P. aeruginosa*, *mexT* encodes the transcriptional activator of *mexEF*-*oprN* that belongs to the LysR family and is located upstream of *mexEF*-*oprN*. Overexpression of MexT activates *mexEF*-*oprN* transcription (27). In *B. pseudomallei*, a similar arrangement exists. The gene encoding the LysR-type regulator BpeT that controls expression of the BpeEF-OprC efflux pump is located upstream of the *llpE-bpeE-bpeF-oprC* operon (Fig. 2A) (30, 31). However, the role of BpeT in control of BpeEF-OprC is not well understood. LysR family proteins can both activate and repress transcription depending on the interaction(s) with coinducer molecules that bind to their carboxy-terminal binding domain (32). The need for coinducer binding may be alleviated by amino acid substitutions in this carboxy-terminal domain allowing constitutive target gene expression.

Because of the pivotal role that SXT plays in treatment and prophylaxis of *B. pseudomallei* infections, the focuses of this study were (i) to determine and characterize the mechanisms leading to decreased SXT susceptibility using attenuated excluded (i.e., excluded from the requirements of the Federal select agent regulations; https://www.selectagents.gov/) select-agent strain Bp82 (33), (ii) to determine the molecular basis of SXT^r^ in a collection of clinical isolates that had evolved resistance during infection, and (iii) to provide a possible explanation as to why primary SXT^r^ is uncommon.

(Portions of this research were conducted by N. L. Podnecky and K. A. Rhodes in partial fulfillment of the requirements for a PhD from Colorado State University, Fort Collins, CO, 2013 and 2016, respectively.)

## RESULTS

### Characterization of TMP-resistant strains.

**FIG 3:**
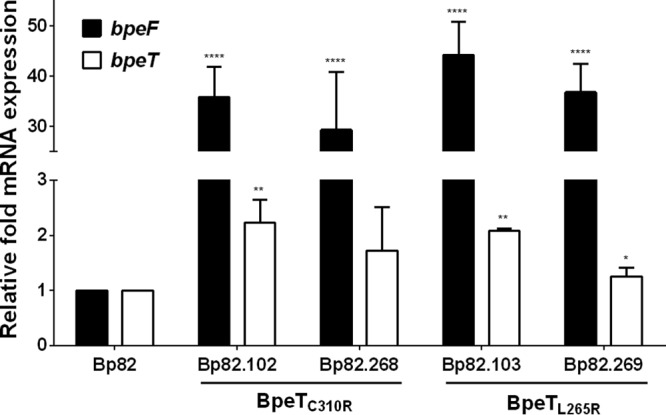
BpeT mutations present in trimethoprim-resistant mutants cause significant increases in* bpeF* mRNA levels. The relative levels of *bpeF* (black bars) and *bpeT* (white bars) expression in Bp82-derived TMP^r^ isolates Bp82.102 and Bp82.103 were determined by reverse transcription-quantitative PCR. BpeT mutations originally found in Bp82.102 and Bp82.103 (BpeT_C310R_ and BpeT_L265_, respectively) were introduced into wild-type Bp82 by allelic replacement of the resident *bpeT* gene, resulting in strains Bp82.268 and Bp82.269, respectively. All expression values are relative to Bp82. Error bars indicate the standard deviation of comparisons between three biological replicates, each of which was performed in technical triplicate. Statistical analysis was done by two-way ANOVA and Tukey’s multiple-comparison test. ****, *P* < 0.0001; **, *P* < 0.01; *, *P* < 0.05.

Following passive selection of Bp82 in the presence of TMP, a collection of isolates with decreased TMP susceptibilities was obtained. Of these, 3 isolates with TMP MICs above the detection limit (≥32 μg/ml), Bp82.102, Bp82.103, and Bp82.104, were selected for further testing and characterization (see Table S1 in the supplemental material). Isolates Bp82.102 and Bp82.103 had over 10-fold increases in SXT MICs, but the observed values were below the 4 μg/ml SXT^r^ cutoff for resistance (Table 1). In addition to the folate pathway inhibitors, these two strains also had increased drug MICs for several known substrates of the BpeEF-OprC efflux pump, including acriflavine and chloramphenicol, which exhibited a consistent 2-fold increase and 8-fold increase in MIC, respectively. In contrast, susceptibility to the non-BpeEF-OprC substrates erythromycin and gentamicin remained unaltered. However, the Bp82.104 isolate did not exhibit the same changes in antimicrobial susceptibilities. We confirmed that increased expression of the BpeEF-OprC efflux pump contributes to the increased MICs observed in strains Bp82.102 and Bp82.103 using reverse transcription-quantitative PCR (RT-qPCR). Compared to wild-type Bp82, there was a significant increase in *bpeF* (>30-fold) and *bpeT* (~2-fold) mRNA expression in strains Bp82.102 and Bp82.103 (Fig. 3) but no change in Bp82.104 (data not shown). These expression patterns suggest that BpeEF-OprC is responsible for resistance to TMP in Bp82.102 and Bp82.103 but not Bp82.104.

10.1128/mBio.01357-17.6 TABLE S1  *Burkholderia pseudomallei* strains used in this study. Download TABLE S1, PDF file, 0.1 MB. Copyright © 2017 Podnecky et al. 2017 Podnecky et al. This content is distributed under the terms of the Creative Commons Attribution 4.0 International license.

**TABLE 1:** MICs of antimicrobials for strain Bp82 and TMP- and SXT-resistant mutant derivatives

Strain	MIC (µg/ml)^a^						
	Folate inhibitor			BpeEF-OprC substrate		Nonsubstrate	
	TMP	SMX	SXT	ACR	CHL	ERY	GEN
Bp82	0.75	4	0.094	32	16	256	256
Bp82.102	≥32	ND^b^	1.500	64	128	256	256
Bp82.103	≥32	ND	1.000	64	128	512	256
Bp82.104	≥32	ND	0.125	32	8	128	256
Bp82.191	≥32	≥1,024	4	128	>128	128	64
Bp82.193	≥32	≥1,024	3	128	>128	128	128
Bp82.199	≥32	≥1,024	2	128	>128	256	64
Bp82.202	≥32	≥1,024	6	128	>128	128	128
Bp82.204	≥32	≥1,024	4	128	>128	128	128
Bp82.207	≥32	≥1,024	2	128	>128	128	64

a Trimethoprim (TMP), sulfamethoxazole (SMX), and SXT (SMX plus TMP) MICs were determined by Etest; acriflavine (ACR), chloramphenicol (CHL), erythromycin (ERY), and gentamicin (GEN) MICs were determined by microdilution. Etest detection limits: 1,024 μg/ml for SMX and 32 μg/ml for TMP. Microdilution was not tested above 128 μg/ml for CHL.

b ND, not determined because strain was not SXT^r^.

### *bpeT* mutations cause BpeEF-OprC overexpression and TMP resistance.

**FIG 4:**
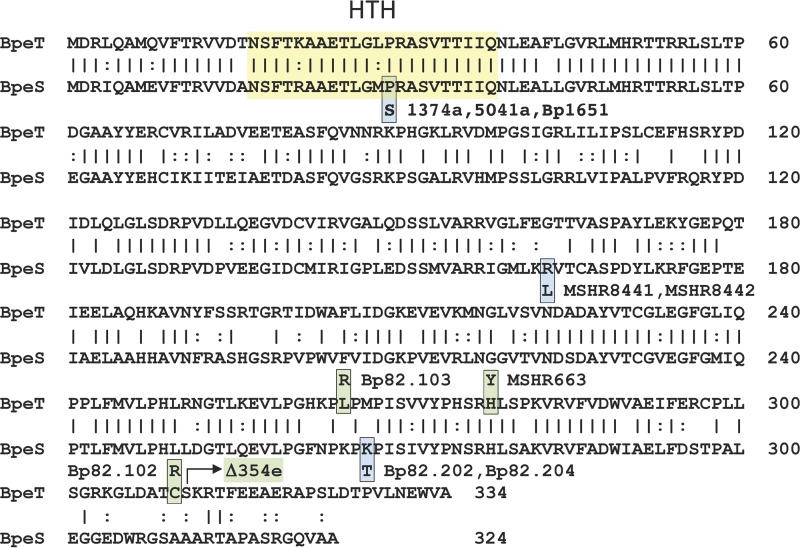
Amino acid sequence homology of BpeT and BpeS and location of mutations in laboratory-selected Bp82 and clinical TMP- and SXT-resistant isolates. Identical amino acids are indicated with vertical lines and similar amino acids with colons. The predicted (Rhone-Alpes bioinformatic Pole Gerland site at https://npsa-prabi.ibcp.fr/cgi-bin/npsa_automat.pl?page=/NPSA/npsa_server.html) helix-turn-helix (HTH) DNA-binding domains of both proteins are framed by the yellow box. BpeT and BpeS mutants are color coded, where green boxes indicate BpeT mutants and blue boxes BpeS mutants. Amino acid substitutions found and confirmed in laboratory-selected mutants are BpeT_C310R_ and BpeT_L265R_ in TMP^r^ mutants Bp82.102 and Bp82.103, respectively, and BpeS_K267T_ in SXT^r^ mutants Bp82.202 and Bp82.204. BpeT_H278T_ and BpeS_R163L_ are presumptive BpeT and BpeS regulatory mutations found in Australian MSHR663, MSHR8441, and MSHR8442 clinical isolates, respectively. "Δ354e" (labeled with an arrow) indicates a truncation of BpeT after amino acid 310 by deletion of the carboxy-terminal 24 codons due to an 800-kb chromosomal inversion in multidrug-resistant Thai clinical isolate 354e (30). The P29S notation denotes the amino acid substitution found in the predicted BpeS HTH region of clinical SXT^r^ isolates 1374a, 5041a, and Bp1651.

To determine if changes to *bpeT* were the cause of the observed overexpression of the *bpeEF*-*oprC* operon and of *bpeT* in Bp82.102 and Bp82.103, the *bpeT* gene was PCR amplified and sequenced. We identified two single nucleotide polymorphisms (SNPs) in *bpeT* resulting in amino acid substitutions in the carboxy-terminal domain of BpeT, namely, C310R in Bp82.102 and L265R in Bp82.103 (Fig. 4). Strain Bp82.104 contained no *bpeT* mutations. To confirm that these *bpeT* mutations cause overexpression of *bpeF* and *bpeT* mRNA, each mutation was individually introduced into the Bp82 wild-type background by allele replacement. The resulting strains, Bp82.268 (BpeT_C310R_) and Bp82.269 (BpeT_L265R_), had *bpeF* mRNA expression profiles similar to those found in the original TMP^r^ isolates (Fig. 3). MIC testing of Bp82.268 and Bp82.269 showed a greater than 5-fold increase in TMP MICs (from 0.75 μg/ml in Bp82 to 4 μg/ml in Bp82.268 and Bp82.269) (Table 2). However, this is below the ≥32 μg/ml observed in the original Bp82.102 and Bp82.103 strains. There were also a 4-fold increase in SXT MICs and a small 2-fold increase in SMX MICs (Table 2) that were again below those observed in the originally selected Bp82.102 and Bp82.103 strains. These data suggest that (i) both BpeT amino acid changes (C310R and L265R) result in increased expression of the BpeEF-OprC efflux pump, which in turn results in TMP resistance and reduced susceptibility to SMX and SXT; (ii) the BpeT mutations do promote *bpeEF-oprC* expression and resistance, not as a result of increased BpeT expression but rather because they likely activate BpeT such that gene expression occurs independently of coeffector binding; and (iii) factors other than mutations in *bpeT* contribute to the increased TMP, SMX, and SXT resistance levels observed in the selected Tmp^r^ strains.

**TABLE 2:** Mutations to BpeT or FolA contribute to increased TMP resistance

Strain	Mutation	MIC (μg/ml)		
		TMP	SMX	SXT
Bp82	None	0.75	4	0.094
Bp82.268	BpeT_C310R_	4	8	0.38
Bp82.269	BpeT_L265R_	4	8	0.38
Bp82.183	FolA_F158V_	24	ND^a^	0.5
Bp82.184	FolA_I99L_	≥32	ND	0.5

a ND, not determined because strain was not SXT^r^.

### Dihydrofolate reductase mutations contribute to TMP resistance.

TMP^r^ strain Bp82.104 did not contain any *bpeT* mutations and did not overexpress BpeEF-OprC. To identify other potential mechanisms of resistance, the *folA* gene encoding DHFR, the TMP drug target (Fig. 1 and 2D), was examined. DNA sequencing of Bp82.104 *folA* revealed a single SNP resulting in an I99L amino acid substitution. The putative involvement of this mutation in TMP^r^ in clinical isolate Bp1651 was previously reported, but the involvement was not experimentally verified (34). While Bp82.103 had no changes in the *folA* sequence, the Bp82.102 *folA* gene also contained a mutation causing an F158V change. Thus, strain Bp82.102 had mutations in both *bpeT* and *folA*. Allelic replacement was used to introduce each *folA* SNP singly into Bp82, and TMP and SXT MICs were determined for each of the resulting strains, Bp82.183 (*folA*_F158V_) and Bp82.184 (*folA*_I99L_). Both *folA* mutations caused TMP^r^ in the engineered mutant strains (Table 2). There was an over 10-fold increase in the SXT MICs (from 0.094 µg/ml to 0.38 µg/ml), but the data from these isolates did not meet the cutoff for SXT^r^, suggesting that *folA* modification alone may not be sufficient for SXT^r^.

To assess the propensity for selection of *folA* mutations in the absence of BpeEF-OprC-mediated efflux, we also selected for TMP^r^ in strain Bp82 Δ(*bpeEF-oprC*) or strain Bp82 Δ*bpeT*. Twelve resulting TMP^r^ isolates capable of growth on 16 µg/ml TMP were sequenced to identify *folA* mutations. All had the FolA_I99L_ mutation that was identified in Bp82.104, possibly due to clonal expansion (data not shown). Together, these findings suggest that *folA* target mutations may be a common cause of or contributor to acquired TMP^r^ in the presence or absence of BpeEF-OprC efflux pump expression or in other strongly selective environments.

### BpeT is a transcriptional activator of *bpeEF-oprC.*

**FIG 5:**
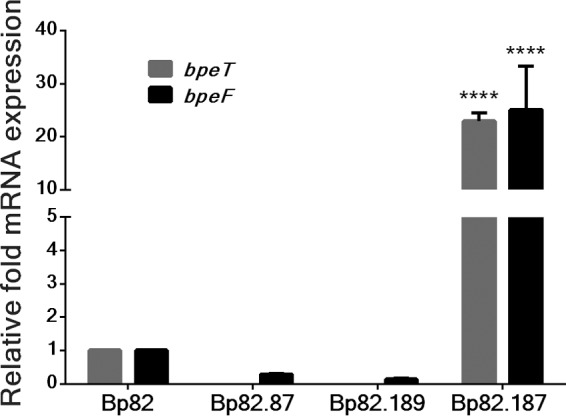
BpeT is a transcriptional activator of *bpeEF-oprC*. A mini-Tn*7* element containing a *bpeT* gene whose expression is driven by the constitutive *P1* promoter was integrated into the genome of Bp82.87 (Bp82 Δ*bpeT*) to create Bp82.187. The Bp82.189 control strain was created by introducing the empty mini-Tn*7* vector into the same strain background. The *bpeF* (black bars) and *bpeT* (gray bars) mRNA levels were determined by RT-qPCR and normalized to expression in Bp82. Error bars represent standard deviations of mean expression levels. Results of statistical analysis performed by one-way ANOVA and Tukey’s multiple-comparison test show that *bpeF* and *bpeT* expression in response to *bpeT* overexpression was significantly different from that seen with Bp82, its Δ*bpeT* derivative (Bp82.87), and empty vector (Bp82.189) controls. ****, *P* < 0.0001.

BpeEF-OprC was previously shown to cause resistance to TMP (28), as was also observed in this study; however, the role of *bpeT* in transcriptional control of this efflux pump is not well understood. The genetic organization and substrate specificity of the *P. aeruginosa* MexEF-OprN efflux pump, which is positively regulated by the LysR-type regulator MexT, and those of BpeEF-OprC are similar, which led us to suggest that they may be regulated in analogous fashions (35). We hypothesized that BpeT overexpression would cause activation of *bpeEF-oprC* transcription and thus would reduce the susceptibility of *B. pseudomallei* to BpeEF-OprC substrates such as TMP. To test this, *bpeT* was cloned behind the constitutive *P1* promoter on a mini-Tn*7* element, which was integrated into the genome of a Δ*bpeT* Bp82 strain derivative, Bp82.87, to yield Bp82.187. This strain expressed *bpeT* and *bpeF* mRNA at levels that were over 20-fold higher than those seen with wild-type Bp82 (Fig. 5). Overexpression of *bpeEF*-*oprC* in this strain resulted in TMP^r^ and reduced SMX and SXT susceptibility (Table S2). These data confirm that BpeT is an activator of *bpeEF-oprC* transcription and that reduced TMP and SMX susceptibility is a direct result of increased BpeEF-OprC expression. However, the modest increase in SMX resistance bestowed by BpeEF-OprC expression is not sufficient to cause SXT^r^.

10.1128/mBio.01357-17.7 TABLE S2  Antimicrobial susceptibilities resulting from BpeT and BpeS overexpression. Download TABLE S2, PDF file, 0.1 MB. Copyright © 2017 Podnecky et al. 2017 Podnecky et al. This content is distributed under the terms of the Creative Commons Attribution 4.0 International license.

### Decreased SXT susceptibility is dependent on expression of BpeEF-OprC.

Mechanisms of SMX resistance in *B. pseudomallei* have not been described, and several attempts to select for SMX resistance in Bp82 failed for unknown reasons. We instead focused on identifying mechanisms of resistance to the SXT combination to infer potential factors affecting SMX susceptibility. Following serial passage of Bp82 in increasing concentrations of SXT, six strains with decreased SXT susceptibilities were randomly selected for further examination: Bp82.191, Bp82.193, Bp82.199, Bp82.202, Bp82.204, and Bp82.207. These isolates had TMP and SMX MICs above the limits of detection (32 μg/ml and 1,024 μg/ml, respectively) (Table 1). The SXT MICs ranged from 2 μg/ml to 6 μg/ml, and three of the isolates (Bp82.191, Bp82.202, and Bp82.204) were classed as resistant to SXT (MIC, ≥4 μg/ml). The drug MICs of SXT mutants were also increased for the known BpeEF-OprC substrates tested, acriflavine and chloramphenicol, although susceptibilities to erythromycin were unchanged and gentamicin susceptibilities were reduced 2-fold to 4-fold (Table 1). Deletion of the *bpeEF-oprC* genes resulted in a major drop in the TMP, SMX, and SXT MICs (Table S3), and subsequent single-copy complementation performed with the wild-type *bpeEF-oprC* operon expressed by the *P*_*tac*_ promoter caused increases in the MICs for TMP, SMX, and SXT, though not to the levels observed with the original isolates (Table S3). A possible explanation is that *P*_*tac*_ may not give the same high-level BpeEF-OprC expression as the native promoter in the presence of the *bpeS* mutations contained in these strains. These data show that the BpeEF-OprC efflux pump plays a significant role in SXT resistance.

10.1128/mBio.01357-17.8 TABLE S3  The BpeEF-OprC efflux pump is required for increased folate inhibitor resistance in laboratory strains and clinical isolates. Download TABLE S3, PDF file, 0.1 MB. Copyright © 2017 Podnecky et al. 2017 Podnecky et al. This content is distributed under the terms of the Creative Commons Attribution 4.0 International license.

### Overexpression of BpeEF-OprC in SXT-resistant isolates is BpeT independent.

**FIG 6:**
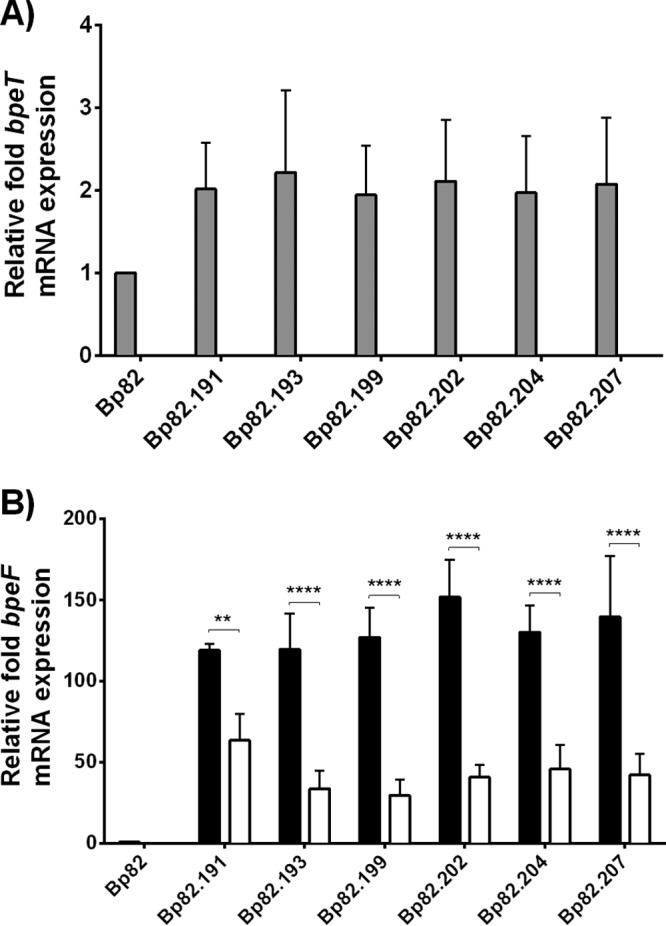
Relative *bpeF* and *bpeT* transcript levels determined for Bp82-derived SXT-resistant isolates. Relative *bpeF* and *bpeT* transcript levels were determined in the indicated strains. All fold expression values are relative to the Bp82 parent strain. (A) *bpeT* transcript levels in SXT^r^ strains Bp82.191 to Bp82.207 (gray bars) and the respective Δ*bpeT* derivatives (no detectable *bpeT* transcripts). (B) *bpeF* transcript levels in SXT^r^ strains Bp82.19 1to Bp82.207 (black bars) and the respective Δ*bpeT* derivatives (open bars). Error bars indicate the standard deviations determined for three biological replicates. Statistical analysis was done by two-way ANOVA and Tukey’s multiple-comparison test. ****, *P* < 0.0001; **, *P* < 0.01.

RT-qPCR was used to determine the relative expression levels of *bpeT* and *bpeF* mRNA in the six isolates (Bp82.191 to Bp82.207) with reduced SXT susceptibility both with and without the BpeT transcriptional regulator. Expression of *bpeT* mRNA was detected in all six isolates relative to Bp82, but intraisolate *bpeT* expression level differences were not statistically significant (Fig. 6A). Remarkably, each of the isolates had *bpeF* mRNA levels that were over 120 times higher than those seen with Bp82 (Fig. 6B). The *bpeF* mRNA expression levels were also determined in derivatives lacking *bpeT*. We observed a significant decrease in *bpeF* mRNA expression in the absence of *bpeT*, but *bpeF* mRNA was still overexpressed by at least 30-fold relative to Bp82 (Fig. 6B). This suggests that, while BpeT enhances expression of the *bpeEF*-*oprC* operon and BpeT overexpression causes constitutive BpeEF-OprC expression and decreased susceptibilities to several antimicrobials, it is neither essential for expression nor the only transcriptional regulatory component for this efflux pump.

### SXT-resistant isolates contain mutations in *bpeS* encoding a novel LysR-type regulator and *folM.*

Using targeted DNA sequencing of strains Bp82.191 to Bp82.207, we found no mutations in *bpeT* or in the *bpeT-llpE* intergenic region, which contains predicted regulatory sequences for both *bpeT* and the *llpE*-*bpeE-bpeF*-*oprC* operon (Fig. 2A). Additionally, we did not find any mutations in the *folA* and *folP* genes, encoding the targets for TMP and SMX, respectively (Fig. 2D and E). Whole-genome sequencing of Bp82.202 and Bp82.204 and comparison to the Bp82 parent revealed two SNPs present in both of the mutants. The first mutation was found in *BP1026B*_*I2790* (*BPSL0731*; strain K96243 annotation) (Fig. 2B), a gene annotated as encoding a LysR-type transcriptional regulator (named BpeS here because of its high similarity to BpeT). BpeS (324 amino acids) showed 61.7% identity overall to the 334-amino-acid BpeT protein (Fig. 4). This identity level increases to 90% over the first 60 amino-terminal amino acids, including the respective predicted helix-turn-helix DNA-binding domains, which are 90% identical and 100% similar. This suggests that BpeT and BpeS likely bind to similar regulatory sequences. The mutation to *bpeS* results in an amino acid change, K267T, in the putative carboxy-terminal effector-binding domain of the regulator.

The second SNP was found in the *BP1026B*_*II0040* (*BPSS0039*; strain K96243 annotation) gene, which is annotated as *ptr1* (pteridine reductase 1). This gene likely corresponds to the *folM* gene found in other bacteria and is referred to as *folM* from this point on (Fig. 2C). The mutation causes a V15G amino acid change in the amino-terminal domain of FolM. This amino acid change lies within the predicted NADPH-binding site consensus sequence of the protein and thus likely affects its activity (see Fig. S1 in the supplemental material), although this has yet to be shown.

10.1128/mBio.01357-17.2 FIG S1  FolM gene and protein mutations in laboratory-selected Bp82 and clinical SXT-resistant isolates. Download FIG S1, PDF file, 0.5 MB. Copyright © 2017 Podnecky et al. 2017 Podnecky et al. This content is distributed under the terms of the Creative Commons Attribution 4.0 International license.

Both the BpeS_K267T_ and FolM_V15G_ mutations were confirmed in strains Bp82.202 and Bp82.204 by targeted Sanger sequencing of PCR-amplified DNA fragments containing the respective mutant genes. Additionally, DNA sequencing of these genes showed that all six SXT-selected isolates—Bp82.191 to Bp82.207—contained the BpeS_K267T_ mutation and that all except Bp82.193 contained the FolM_V15G_ mutation. Bp82.193 instead had a single base deletion at *folM* nucleotide position 203 causing a frameshift mutation after amino acid 67 and early termination of the protein following residue 92 (Fig. S1). Bp82.193 is phenotypically similar to the other strains in all aspects, supporting the notion that the the FolM_V15G_ mutation may indeed deleteriously affect protein function.

### Mutations in *bpeS* and *folM* contribute to decreased SXT susceptibility.

To confirm the role of the identified mutations in *bpeS* and *folM* in decreased SXT susceptibility, the *bpeS*_K267T_ and *folM*_V15G_ mutations were repaired individually and in combination in strains Bp82.202 and Bp82.204. Repair of *bpeS*_K267T_ resulted in *bpeS*-positive (*bpeS*^+^) strains Bp82.246 and Bp82.249; repair of *folM*_V15G_ resulted in *folM*^+^ strains Bp82.247 and Bp82.250; and repair of both *bpeS*_K267T_ and *folM*_V15G_ resulted in *bpeS*^+^ *folM*^+^ strains Bp82.248 and Bp82.251. MIC testing indicated that loss of either SNP differentially restored susceptibility to TMP, SMX, and SXT (Table 3). Repair of the *bpeS* gene (Bp82.246 and Bp82.249) caused a greater reduction in MIC, where the susceptibilities of the repaired mutants matched that of the parental strain, Bp82. Repair of the *folM* gene (Bp82.247 and Bp82.250) reduced the SMX MIC from the detection limit of 1,024 μg/ml to 32 μg/ml and the SXT MIC from 4 to 6 μg/ml to 0.75 μg/ml but did not affect the TMP MIC, which remained above the ≥32 μg/ml detection limit. For unknown reasons, repair of both SNPs (Bp82.248 and Bp82.251) resulted in MICs below those of the original Bp82 parent strain.

**TABLE 3:** Antimicrobial susceptibilities of genetically repaired co-trimoxazole-resistant Bp82 isolates

Strain and relevant genotype	MIC (μg/ml)		
	TMP	SMX	SXT
Bp82 *bpeS*_WT_ *folM*_WT_	0.75	4	0.047
Bp82.202 *bpeS*_K267T_ *folM*_V15G_	≥32	≥1,024	6
Bp82.204 *bpeS*_K267T_ *folM*_V15G_	≥32	≥1,024	4
Bp82.246 *bpeS*_WT_ *folM*_V15G2_^a^	0.5	4	0.064
Bp82.249 *bpeS*_WT_ *folM*_V15G_^b^	0.5	4	0.064
Bp82.247 *bpeS*_K267T_ *folM*_WT_^a^	≥32	32	0.750
Bp82.250 *bpeS*_K267T_ *folM*_WT_^b^	≥32	32	0.750
Bp82.248 *bpeS*_WT_ *folM*_WT_^a^	0.19	1.5	0.032
Bp82.251 *bpeS*_WT_ *folM*_WT_^b^	0.19	1.5	0.032

a Derived from Bp82.202.

b Derived from Bp82.204.

**FIG 7:**
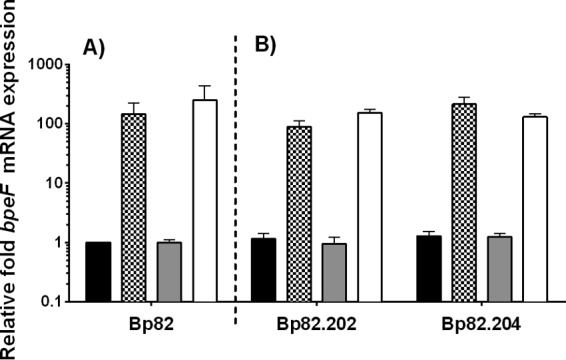
The BpeS_K267T_ mutation is responsible for increased *bpeEF-oprC* gene expression in SXT^r^ strains. The relative levels of *bpeF* mRNA expression in Bp82 derived-isolates were determined by RT-qPCR. Bp82.202 and Bp82.204 are SXT^r^ strains, with each possessing an SNP in both *bpeS* and *folM*. (A) The *bpeS*_K267T_ (checkered bars), *folM*_V15G_ (gray bars), and *bpeS*_K267T_ plus *folM*_V15G_ (open bars) SNPs were sequentially introduced into wild-type Bp82 (black bars). (B) The same SNPs were repaired individually and in combination in Bp82.202 and Bp82.204. Black bars indicate the wild type, i.e., repair of both *bpeS*_K267T_ and *folM*_V15G_; checkered bars indicate the repair of *folM*_V15G_ but the presence of *bpeS*_K267T_; gray bars indicate the repair of *bpeS*_K267T_ but the presence of *folM*_V15G_; open bars indicate no repair and the presence of both *bpeS*_K267T_ and *folM*_V15G_. All fold expression values are relative to Bp82. Error bars indicate the standard deviations determined from comparisons between three biological replicates. Isolates containing the BpeS_K267T_ mutation expressed *bpeF* at very high levels.

BpeEF-OprC expression was analyzed in strains engineered to contain *bpeS*_K267T_ or *folM*_V15G_ or both. Introduction of *bpeS*_K267T_ into *bpeS*_WT_ strain Bp82 resulted in >100-fold increased expression of *bpeF*, and, as expected, there was no change in *bpeF* expression seen with the introduction of the *folM*_V15G_ mutation (Fig. 7A). The same *bpeF* expression patterns were observed in Bp82.202 and Bp82.204 with neither mutation repaired and in their derivatives that contain only the *folM*_V15G_ mutation (Bp82.247 and Bp82.250) (Fig. 7B).

### BpeS is a conditional transcriptional activator of BpeEF-OprC.

**FIG 8:**
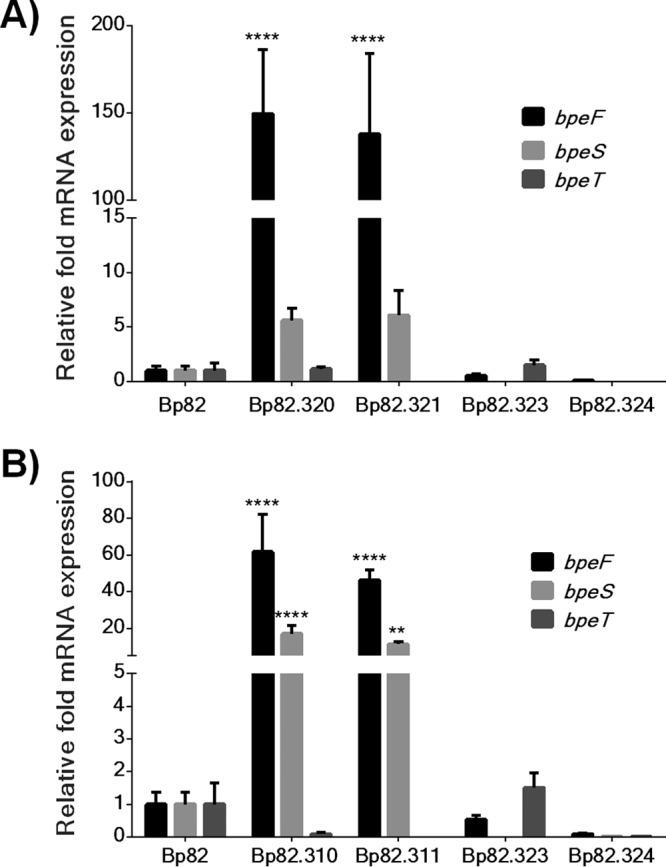
BpeS is a conditional transcriptional activator of BpeEF-OprC. Empty mini-Tn*7* vectors or constructs containing the *bpeS* alleles indicated below were introduced into Bp82 derivatives lacking *bpeS* (Bp82.264) or lacking *bpeS* and *bpeT* (Bp82.286). The expression levels of *bpeS*, *bpeT*, and *bpeF* in the resulting strains were measured by RT-qPCR, and two-way ANOVA was performed with Dunnet's test to determine significance compared to the Bp82 control. Error bars represent standard deviations of the mean levels of expression for at least two biological replicates. ****, *P* < 0.0001; **, *P* < 0.01. (A) Expression of *bpeS*_K267T_. Strains overexpressing BpeS_K267T_, Bp82.320 (Δ*bpeS* background), and Bp82.321 (Δ*bpeS* Δ*bpeT* background) significantly overexpressed *bpeF* mRNA. *bpeF* transcript levels were significantly elevated regardless of the presence or absence of *bpeT*. Although *bpeS* expression was increased ~5-fold to ~7.5-fold in these isolates, the levels were not statistically significantly different from those seen with the Bp82 control. *bpeT* levels were not affected by the presence of mutant *bpeS* in Bp82.320. The presence of the empty vector in Bp82.323 (Δ*bpeS* background) and Bp82.324 (Δ*bpeS* Δ*bpeT* background) had no significant effect on *bpeF* transcription. (B) Expression of *bpeS*_P29S_. Strains overexpressing BpeS_P29S_, Bp82.320 (Δ*bpeS* background), and Bp82.321 (Δ*bpeS* Δ*bpeT* background) demonstrated significant overexpression of *bpeF* and *bpeS*. Strains Bp82.310 and Bp82.320 with *P1*-*bpeS*_P29S_ displayed elevated expression of *bpeS* and *bpeF* mRNA but not *bpeT* mRNA. The presence of empty vector in strain Bp82.323 (Δ*bpeS* background) or strain Bp82.324 (Δ*bpeS* Δ*bpeT* background) had no effect on *bpeF* transcription; however, *bpeT* levels were slightly increased in Bp82.323, but this expression was not statistically significantly different from that seen with Bp82.

Due to the high degree of similarity between BpeT and BpeS, we assessed the role of BpeS in BpeEF-OprC expression specifically via activation of *llpE-bpeE-bpeF-oprC* operon transcription. To this end, mini-Tn*7* vectors were constructed where *bpeS* was constitutively expressed from the *P1* promoter. The mini-Tn*7*-*P1*-*bpeS* construct was transposed into Δ*bpeS* strain Bp82.264 to create Bp82.289 or into Δ*bpeS* Δ*bpeT* strain Bp82.286 to create Bp82.288. RT-qPCR analyses indicated that *bpeS* mRNA levels in these strains were increased ~12-fold compared to the Bp82 results (data not shown). However, this BpeS overexpression affected neither the expression of *bpeF* nor the host strain’s susceptibility to TMP, SMX, or SXT (Table S2). In contrast, a Δ*bpeS* strain containing a chromosomally inserted mini-Tn*7*-*P1*-*bpeS*_K267T_ construct (Bp82.320) and a Δ*bpeS* Δ*bpeT* strain containing the same construct (Bp82.321) overexpressed BpeEF-OprC with *bpeF* mRNA levels >100-fold higher than those seen with Bp82 (Fig. 8A). This overexpression of BpeEF-OprC resulted in TMP, SMX, and SXT resistance (Table S2). These results corroborate the findings described above in that both TMP and SMX are substrates of BpeEF-OprC. They also suggest that altered BpeS function (i.e., activation of *bpeEF-oprC* transcription independently of the presence of a coinducer), rather than an increase in *bpeS* transcription, is responsible for differential levels of expression of the *llpE-bpeE-bpeF-oprC* operon.

### SXT-resistant clinical isolates contain *folM* and BpeEF-OprC regulatory mutations.

SXT resistance in clinical isolates is rare but can occasionally occur (23, 24, 36). To assess whether any of the genes mutated in laboratory-selected SXT^r^ strain Bp82 derivatives also contained mutations in SXT^r^ clinical isolates, we analyzed genes of the few SXT^r^ strains identified to date by targeted sequencing or genome sequencing or both. Strain pair 354b (SXT^s^) and 354e (SXT^r^) represents two sputum isolates obtained from the same patient 75 months apart (30, 31). Phenotypically, these strains differ in that the 354e relapse isolate exhibits a multidrug resistance phenotype consistent with BpeEF-OprC expression, likely due to the observed truncation of BpeT (30, 31) (Fig. 4). Strain 354e also contains a previously noted 7-bp deletion in *folM* which frameshifts FolM after amino acid 20 (30) (Fig. S1). Strains MSHR664 (SXT^s^) and MSHR663 (SXT^r^) are sequential isolates obtained from a relapse melioidosis patient in Darwin, Australia (37). In addition to an SNP that causes an H278Y amino acid substitution in BpeT (Fig. 4), MSHR663 contains the same 7-bp deletion in *folM* that is present in SXT^r^ Thai isolate 354e (Fig. S1). Australian strains MSHR8441 and MSHR8442 are SXT^r^ isolates from a cystic fibrosis patient (36, 38). Although not noted at the time, both MSHR8441 and MSHR8442 contain an SNP causing an R163L amino acid substitution in BpeS in comparison to 1026b (Fig. 4) and a 9-bp in-frame insertion in *folM* (Fig. S1). The latter mutation duplicates FolM amino acids 20 to 22. Lastly, strain 5041a is an SXT^r^ sputum isolate from a Thai melioidosis patient. The *folM* gene of 5041a contains the same 7-bp deletion present in strains MSHR663 and 354e. Compared to several SXT^s^ strains, a single unique SNP in *bpeS* was identified in 5041a that caused a P29S amino acid substitution in the putative helix-turn-helix DNA-binding domain of BpeS (Fig. 4). Of note is that two SXT^r^ strains, 1374a from Thailand (23, 24) and Bp1651 from Australia (34), did not contain any *bpeT* or *folM* mutations but rather only the *bpeS*_P29S_ allele. Several lines of evidence indicate that the BpeS P29S substitution plays a role in BpeEF-OprC expression and thus in SXT resistance. First, this mutation is present in multiple SXT^r^ strains, including 5041a and 1374a from Thailand and Bp1651 from Australia. Second, we observed by RT-qPCR that *bpeF* is highly expressed in 5041a and 1374a, i.e., at levels that are 78-fold and 118-fold higher, respectively, than that seen with 1026b; a similar high level of expression was observed in a Bp82 derivative, Bp82.284, which contains chromosomal *bpeS*_P29S_ (Fig. S2). Third, overexpression of *bpeS*_P29S_ in a Bp82 Δ*bpeS* strain (Bp82.320) or a Bp82 Δ*bpeT* Δ*bpeS* strain (Bp82.321) led to high-level *bpeF* mRNA expression (Fig. 8B) and SXT resistance (Table S2). Fourth, a *bpeEF-oprC* deletion abolishes TMP^r^, SMX^r^, and SXT^r^ in both strain 1374a and strain 5041a (Table S3). These results further support the notion that altered BpeS function and not an increase in protein levels is responsible for the observed changes in BpeEF-OprC expression levels.

10.1128/mBio.01357-17.3 FIG S2  Clinical and laboratory strains containing chromosomal *bpeS*_P29S_ express high levels of *bpeF*. Download FIG S2, PDF file, 0.2 MB. Copyright © 2017 Podnecky et al. 2017 Podnecky et al. This content is distributed under the terms of the Creative Commons Attribution 4.0 International license.

## DISCUSSION

*B. pseudomallei* is intrinsically resistant to numerous antimicrobials; however, it is generally susceptible to drugs used in the treatment of melioidosis, including the tetrahydrofolate biosynthetic pathway inhibitor combination SXT (9, 10). SXT is the drug of choice for eradication-phase melioidosis treatment and postexposure prophylaxis of accidental laboratory exposures (9, 11). SXT-resistant isolates are rare in nature but are of concern as SXT resistance significantly reduces available treatment options (23). The main purposes of this study were to explore and characterize the molecular mechanisms responsible for antimicrobial resistance to the clinically relevant tetrahydrofolate pathway inhibitors TMP and SMX and to better understand why SXT^r^ resistance is uncommon in *B. pseudomallei*.

Our findings illustrate that the nature of resistance to SXT in *B. pseudomallei* is complex and does not fit the pattern typically seen in other SXT^r^ bacteria. Typically, bacterial resistance to TMP and SMX, the two components of SXT, is the result of mutations to the DHFR (FolA) and DHPS (FolP) drug targets (Fig. 1) (12, 20). While we have shown that mutation of FolA alone can cause high-level TMP^r^ in laboratory-selected mutants, SMX resistance due to *folP* mutations could not be demonstrated. This finding is paralleled by observations made with clinical isolates where the presence of FolA mutations, e.g., the FolA_I99L_ mutation in SXT^r^ Australian strain Bp1651 (34), had previously been implicated in TMP^r^ but where *folP* mutations have yet to be identified. In this study, we showed that SXT^r^ in *B. pseudomallei* employs a novel strategy of regulation of BpeEF-OprC efflux pump expression that employs two closely related LysR-type transcriptional regulators, BpeT and BpeS. Further contributing to SXT^r^ is FolM, a folate pathway-associated protein whose function is not yet well understood and which has not been previously been implicated in folate inhibitor resistance.

*In vitro* exposure to either TMP or SXT selects for regulatory mutants that constitutively express BpeEF-OprC. However, the natures of the regulatory mutations and the levels of efflux pump expression selected by the two drugs are different and this differential regulation of expression determines the drug resistance profile. Exposure to TMP alone selected for mutations affecting the putative carboxy-terminal effector-binding domain of BpeT. These are likely activating mutations that relieve the protein’s dependence on interaction with an unknown coinducer, possibly a pump substrate. Constitutive BpeEF-OprC expression in the TMP^r^ isolates was accompanied by differences in the SMX MICs, but this level of expression is insufficient to cause SMX^r^ and thus to cause clinically significant SXT^r^. Exposure to increasing concentrations of SXT led to SXT resistance. This resistance is largely due to constitutive overexpression of BpeEF-OprC, likely by activated mutant BpeS. The underlying mutations affect either the carboxy-terminal domain or the amino-terminal helix-turn-helix DNA-binding domain of BpeS. The constitutive overexpression of the BpeEF-OprC efflux pump mainly confers TMP resistance but also confers resistance to SMX and thus to SXT.

Despite that these results clearly implicate BpeT and BpeS in regulation of *bpeEF-oprC* gene expression, at present we do not fully understand the involvement of two LysR-type regulators in regulation of BpeEF-OprC expression. What we do know is that neither regulator is essential for control of *bpeEF-oprC* gene expression. If either *bpeT* or *bpeS* were an essential activator of pump gene expression, loss of one or both regulatory genes would result in abrogated pump expression, which is not what we observed in induction studies. These studies showed that *bpeF* expression was induced to similar levels in the wild type and in a *bpeS bpeT* double mutant by the BpeEF-OprC substrates chloramphenicol and doxycycline (see Fig. S3 in the supplemental material). Wild-type-like induction in the absence of both BpeT and BpeS suggests that neither regulator is solely responsible for expression of *bpeEF-oprC*. These data support the notion that an additional regulatory factor is responsible for substrate-mediated pump induction.

10.1128/mBio.01357-17.4 FIG S3  BpeS and BpeT are dispensable for expression of *bpeEF-oprC* in the presence of some pump substrates. Download FIG S3, PDF file, 1.4 MB. Copyright © 2017 Podnecky et al. 2017 Podnecky et al. This content is distributed under the terms of the Creative Commons Attribution 4.0 International license.

We have shown that purified BpeT and BpeS, as well as representative mutant derivatives, e.g., BpeT with carboxy-terminal mutations, BpeS_K267T_, and BpeS_P29S_, bind to the same sequence corresponding to the *bpeT-llpE* intergenic region (K. A. Rhodes and H. P. Schweizer, unpublished data). Thus, both regulators and their mutant versions affect transcription of the *llpE*-*bpeE-bpeF-oprC* operon directly, but likely independently, which suggests that they might activate BpeEF-OprC expression in response to different stimuli. This idea is especially interesting when combined with the fact that RND efflux systems such as BpeEF-OprC are adaptation mechanisms needed for response to environmental stressors other than antimicrobials (39–42). The expression of multiple, highly contextual regulatory proteins may allow flexible employment of BpeEF-OprC to manage multiple environmental conditions. The finding that *bpeS* mutations seem to drive greater efflux gene expression than *bpeT* mutations perhaps reflects greater affinity of the respective proteins for the common binding site.

Involvement of several transcriptional regulators in efflux pump expression is well documented. For instance, expression of the MexAB-OprM efflux pump in *P. aeruginosa* is controlled by at least eight regulators, five of which bind in the regulatory region of the *mexAB-oprM* operon (reviewed in reference 43). One of these regulatory proteins, MexT, the LysR-type transcriptional activator of MexEF-OprN, the *P. aeruginosa* homolog of BpeEF-OprC, exerts a negative effect on MexAB-OprM expression, but the underlying mechanism remains largely unknown (44). Such “cross talk” between efflux systems is not uncommon and has been proposed for AmrAB-OprA, BpeAB-OprC, and BpeEF-OprC in *B. thailandensis* although the underlying mechanisms were not investigated (45). Although not explored in this study, the unexpected lowering of gentamicin resistance in the SXT^r^ mutants can possibly be attributed to a negative effect that BpeEF-OprC may exert on AmrAB-OprA expression. However, gentamicin MICs remain well above the clinical breakpoints for susceptibility that would enable potential clinical use of gentamicin and or other aminoglycosides for treating SXT^r^ *B. pseudomallei*.

In addition to BpeS and BpeT, all strains examined in this study (except one) with increased SXT resistance also contained mutations in the annotated *ptr1* gene in *B. pseudomallei*, which is likely *folM* and whose true role in the biology of *B. pseudomallei* and contribution to SXT resistance remain to be established. Although FolM was originally postulated to function as an alternative DHFR (13), subsequent studies suggested that this is not its physiological function. A more likely scenario is that in bacteria expressing FolA, FolM does not function as a DHFR, as dihydrofolate is a poor substrate for purified FolM and its intracellular levels are kept extremely low by FolA (14). The physiological function now attributed to FolM is reduction of dihydromonapterin to tetrahydromonapterin (14) (Fig. 1). In bacteria expressing phenylalanine hydroxylase (PhhA), e.g., *Burkholderia* species, and thus catalyzing the conversion of phenylalanine to tyrosine, terahydromonapterin is a required PhhA cofactor, and FolM is required for its synthesis (14). Tetrahydromonapterin levels often outrank folate levels as an end product of pterin biosynthesis, and tetrahydromonapterin synthesis would establish competition between tetrahydropterin and folate synthesis (14). In this scenario, inactivation of FolM by mutation would increase the level of substrates flowing through the folate biosynthetic pathway, possibly resulting in decreased susceptibilities to folate pathway inhibitors. This notion is supported by the well-established findings showing that hyperproduction of *p*-aminobenzoic acid and increased substrate flux through the folate biosynthetic pathway represent a documented mechanism of sulfonamide resistance (46). Our data are consistent with the notion that mutations that compromise FolM function contribute to but are not alone sufficient to confer TMP, SMX, and SXT resistance.

Our findings determined with laboratory-selected SXT^r^ strains are corroborated by data obtained with clinical *B. pseudomallei* isolates. First, it has been postulated that the previously introduced *B. pseudomallei* SXT^r^ clinical isolate 354e likely expresses BpeEF-OprC due to a truncation of the BpeT carboxy terminus, and this strain has been shown to exhibit an SXT^r^ phenotype (30). Second, SNPs in *bpeS* affecting protein integrity are also found in SXT^r^ clinical isolates. Third, all but one of SXT^r^ clinical *B. pseudomallei* isolates studied to date also possess mutations in *folM*.

In conclusion, our data show that acquired TMP and SXT resistance in *B. pseudomallei* is multifactorial and reflects complex regulation of BpeEF-OprC efflux pump expression and interplay with known and novel folate and pterin pathway constituents. A more complete understanding of these mechanisms will require further studies of the central and yet likely differential roles of BpeS and BpeT in *bpeEF-oprC* gene expression, as well as those of *folM* and its gene product. Knowledge of the factors involved in the SXT resistance seen in *B. pseudomallei* is vital for its rapid identification in clinical settings and for forensic applications. The finding that achieving even modest levels of SXT^r^ requires multiple mutations provides a likely explanation for the rarity of clinically occurring SXT-resistant mutants despite lengthy eradication-phase therapy (23, 24).

## MATERIALS AND METHODS

### Strains and growth conditions.

The attenuated, excluded select-agent Bp82 strain (33) was used for the majority of experiments in this study to avoid concerns respecting dual use of research. Work was performed with Bp82 and its derivatives (see Table S1 in the supplemental material) in biosafety level 2 (BSL-2) facilities at Colorado State University and the University of Florida with approval of the respective institutional biosafety committees. Virulent *B. pseudomallei* strains (Table S1) were handled in select-agent-approved BSL-3 facilities at Colorado State University and the University of Florida. *B. pseudomallei* strains were grown in Lennox Luria Bertani broth or agar (LB or LBA; Mo Bio Laboratories, Inc., Carlsbad, CA) containing 5 g/liter NaCl or cation-adjusted Mueller-Hinton II broth or agar media (MHB or MHA; Becton, Dickinson and Company, Sparks, MD). Bacterial growth medium was supplemented with adenine (Ade; Sigma, St. Louis, MO) for the growth of Bp82 and its derivatives as follows. LB broth or agar was used with 80 μg/ml Ade and MH broth or agar with 40 μg/ml Ade. *E. coli* strains DH5α (47) and RHO3 (48) were used for plasmid DNA manipulation and mobilization, respectively. All cultures were grown at 37°C with aeration, unless otherwise noted.

### Antimicrobial susceptibility testing.

TMP, SMX, and SXT MIC assays were set up using mid-log-phase cells (optical density at 600 nm [OD_600_] = 0.6 to 0.8) following the guidelines provided by the Etest manufacturer (AB BioMérieux, Marcy l’Etoile, France). The MICs of other antibiotics were determined by the standard broth microdilution method, following Clinical and Laboratory Standards Institute (CLSI) guidelines (49). Antimicrobials used for microdilution MIC testing and the respective suppliers were as follows: carbenicillin (CAR; Gemini Bio-Products, West Sacramento, CA) and acriflavine (ACR), chloramphenicol (CHL), erythromycin (ERY), gentamicin (GEN), and SMX and TMP (Sigma-Aldrich Co, St. Louis, MO). All MIC tests were incubated under stationary conditions at 37°C for 16 to 20 h. Where necessary, MICs were also determined in the presence of 1 mM isopropyl-thio-β-d-galactopyranoside (IPTG; Gold Biotechnology, St. Louis, MO) for expression of the *bpeEF-oprC* operon from the inducible *P*_*tac*_ promoter (28). MICs were tested in a minimum of 3 replicates, and final results were reported as the mode of these replicates. Interpretative standards for the Etest were based on CLSI guidelines for broth microdilution, which define SXT (TMP and SMX at a 1:19 ratio) MICs of ≤2/38 μg/ml as susceptible and of ≥4/76 μg/ml as resistant (24, 28, 49). Strains with SXT MICs of ≥4 μg/ml were considered to represent resistance. Because there are no CLSI-established breakpoints for TMP and SMX, we used the following MIC cutoffs to define susceptibility and resistance: for TMP, ≤8 μg/ml for susceptibility and >8 μg/ml for resistance; for SMX, ≤256 μg/ml for susceptibility and >256 μg/ml for resistance (28).

### Passive selection of Bp82 TMP-, SMX-, and SXT-resistant mutants.

TMP^r^ Bp82 mutants were isolated by plating Bp82 on LBA containing 16 μg/ml of TMP (~16 times the MIC). Similarly, spontaneous mutants with reduced SXT susceptibility were selected by serial passage in LB with increasing concentrations of SXT. Briefly, Bp82 was grown overnight and then subcultured at a dilution of 1:100 into LB containing 0.064 μg/ml SXT (~1 times the MIC). The bacteria were successively subcultured into fresh LB with 4-fold increases in SXT, ending at 8 μg/ml SXT. Isolated colonies that grew on LBA with 8 μg/ml SXT were patched onto LBA with 16 μg/ml SXT to confirm the regrowth with SXT.

### Targeted gene sequencing and analysis.

DNA sequencing of specific genes or regulatory regions was performed as previously described (28). Briefly, genomic DNA from selected strains was isolated using PureGene core kit A (Qiagen, Valencia, CA). The target genes were PCR amplified in separate PCRs using platinum *Taq* DNA polymerase High Fidelity (Life Technologies, Inc. Corporation, Carlsbad, CA) and specifically designed primer sets (see Table S4 for the primers used in this study and Text S1 in the supplemental material for details). PCR replicates were pooled and sequenced at the Colorado State University proteomic and metabolomics core facility or the University of Florida Interdisciplinary Center for Biotechnology Research. Alignment of the sequencing reads and subsequent comparisons were performed using Sequencher version 5.1 (Gene Codes Corporation, Ann Arbor, MI).

10.1128/mBio.01357-17.1 TEXT S1  Supplemental methods. Download TEXT S1, PDF file, 0.2 MB. Copyright © 2017 Podnecky et al. 2017 Podnecky et al. This content is distributed under the terms of the Creative Commons Attribution 4.0 International license.

10.1128/mBio.01357-17.9 TABLE S4  Primers used in this study. Download TABLE S4, PDF file, 0.1 MB. Copyright © 2017 Podnecky et al. 2017 Podnecky et al. This content is distributed under the terms of the Creative Commons Attribution 4.0 International license.

### Whole-genome sequencing.

Whole-genome sequencing of Bp82 laboratory strains and clinical isolates was performed by paired-end sequencing using an Illumina GAIIx Genome Analyzer (Illumina, Inc., San Diego, CA) and a Kapa Biosystems library preparation kit (Woburn, MA; catalog number KK8201) protocol with an 8-bp index modification. Details of library preparation, sequencing, and data analysis are provided in Text S1. For single nucleotide polymorphism (SNP) analysis, the sequence read data were aligned to the *B. pseudomallei* 1026b reference genome (NC_017831.1 and NC_017832.1) or *B. pseudomallei* K96243 (NC_006350.1 and NC_006351.1). SNP positions identified were required to have >10× coverage depth and >90% variant base calls.

### Construction of targeted mutants.

The pEXKm5-based allelic replacement system was used for generation of specific gene deletion mutants, repair of SNPs, and introduction of single SNPs into the desired Bp82 or derivative strain background (48). Plasmid-borne marked or unmarked deletion constructs were derived from chromosomal DNA templates of 1026b or of Bp82 and its derivatives and were built by PCR amplification and splicing by overlap extension (SOEing) PCR as detailed in Text S1. Fragments containing the desired mutations were cloned into pEXKm5 (48) (plasmids used in this study are listed in Table S5). The resulting allelic exchange plasmids were conjugated into the target strain using the *E. coli* RHO3 mobilizer strain as previously described (48). Merodiploids were selected on LBA containing 50 μg/ml 5-bromo-4-chloro-3-indoxyl-beta-d-glucuronide (XGluc; Gold Biotechnology, St. Louis, MO) and 300 to 1,000 μg/ml kanamycin (Kan). Merodiploids were resolved by the use of sucrose (MP Biomedicals, Santa Ana, CA) counterselection or, in some cases, both sucrose- and I-SceI-mediated counterselection (48). Flp recombinase target (*FRT*)-flanked Kan or gentamicin resistance markers in deletion mutants were removed by Flp recombinase-mediated excision using the pFLPe2 plasmid or the pFLPe3 plasmid, as previously described (50), resulting in strains containing unmarked deletions. Putative mutants were screened either by PCR to confirm deletion of a target gene or by PCR amplification followed by DNA sequencing to confirm insertion or repair of SNP mutations. Details of plasmid and strain construction are provided in Text S1.

10.1128/mBio.01357-17.10 TABLE S5  Plasmids used in this study. Download TABLE S5, PDF file, 0.1 MB. Copyright © 2017 Podnecky et al. 2017 Podnecky et al. This content is distributed under the terms of the Creative Commons Attribution 4.0 International license.

### Deletion mutant complementation.

Deletion strains were complemented with a *bpeT* or *bpeEF*-*oprC* gene(s) originating from strain 1026b or Bp82 utilizing the mini-Tn*7* system, which allows stable and site-specific single-copy insertions into the *B. pseudomallei* genome at three possible *glmS*-associated *att*Tn*7* sites (50). The respective mini-Tn*7* elements, along with an empty-mini-Tn*7* element used as a control, were transferred to the target *B. pseudomallei* strains either via conjugation from *E. coli* or by electroporation, and *glmS*-associated insertions were verified as previously described (50, 51). Mini-Tn*7* insertions at the *glmS2*-associated *att*Tn*7* site were routinely retained for further studies, unless noted otherwise. The inducible *E. coli trp*/*lac* operon hybrid *P*_*tac*_ promoter was used for regulated expression of the *bpeEF-oprC* genes. BpeEF-OprC expression was induced by addition of 1 mM isopropyl-β-d-thiogalactopyranoside (IPTG; Gold Biotechnology, St. Louis, MO) (28).

### Construction of strains constitutively expressing *bpeS* and *bpeT.*

The *P1* promoter (52) was used for constitutive expression of *bpeT*, *bpeS*, and *bpeS*_K267T_. The *P1-bpeT*, *P1-bpeS*, *P1-bpeS*_K267T_, and *P1-bpeS*_P29S_ constructs were assembled by PCR and cloned into either pUC18T-mini-Tn*7*T-Gm (gentamicin resistance marker) (53) or pUC18T-mini-Tn*7*T-Km (kanamycin resistance marker) (50) as described in Text S1. The recombinant mini-Tn*7* elements were transposed into the chromosomes of the Δ*bpeT* (Bp82.87) strain, the Δ*bpeS* (Bp82.264) strain, or the Δ*bpeT* Δ*bpeS* strain (Bp82.286).

### RNA extraction and reverse transcription-quantitative PCR (RT-qPCR).

Expression levels of mRNA of target genes were analyzed in bacteria grown to mid-log phase (OD_600_ = 0.6 to 0.8) in LB. RNA was isolated using an RNeasy Protect Bacteria minikit (Qiagen, Valencia, CA) in biological triplicate, as previously described (28). Relative expression levels were determined using gene-specific primer sets (Table S4) in technical triplicate. 23S rRNA was used for normalization, and relative fold expression compared to that seen with the parental strains was determined using iCycler iQ Optical System software version 2.0 (Bio-Rad, Hercules, CA) with experimentally defined amplification efficiencies for each primer set. Expression values were pooled between biological replicates, and relative expression data were analyzed by two-way analysis of variance (ANOVA) followed by either Tukey’s multiple-comparison test or Dunnet’s posttest using GraphPad Prism (GraphPad Software, Inc., La Jolla, CA). *P* values of <0.05 were considered significant.

### Data availability.

Whole-genome sequencing data are available from NCBI and published sources, e.g., doi:10.1128/mBio.00356-17, doi:10.1128/genomeA.00254-15, doi:10.1371/journal.pone.0036507, and and NCBI BioProjects PRJNA397943 and PRJNA398084.
