# Supplementary material for: Mechanisms of Resistance to Folate Pathway Inhibitors in Burkholderia pseudomallei: Deviation from the Norm
Source: mBio. 2017 Sep 5;8(5):e01357-17. doi: 10.1128/mBio.01357-17 (PMC5587915; doi:10.1128/mBio.01357-17)
Supplement: FIG S4 [file mbo004173469sf4.pdf]

Figure S4. ***Burkholderia pseudomallei folE*** **encodes a GTP cyclohydrolase I**

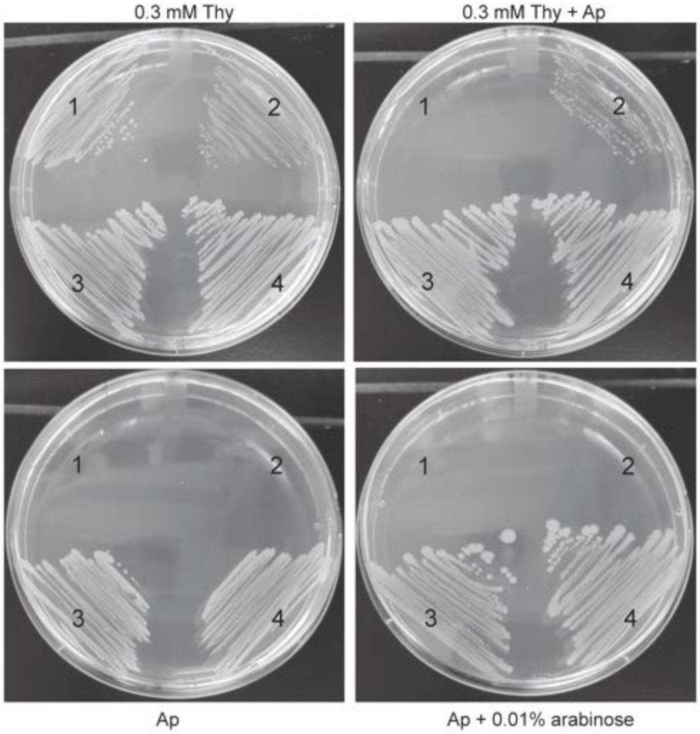

The *folE* gene was PCR amplified from Bp82 genomic DNA using Platinum Taq DNA Polymerase High Fidelity (Life Technologies Corporation, Grand Island, NY) and primers 3077 (5'- ATTGAG**CCCGGG**-CTTCATGGAGTTCAATGCAT; the *Xma*I site is bolded) + 3089 (5'- GCATGA**AAGCTT**GGGTAA-GGTGGCTGGTTT; the *Hin*dIII site is bolded). The resulting 799 bp DNA fragment was TA cloned into pGEM-T Easy (Promega, Madison, WI) to create pPS3360. Next, a 786 bp *Xma*I-*Hin*dIII fragment was excised from pPS3360 and ligated between the same sites of pHERD20T (**Qiu D** et al. 2008. Appl Env Microbiol **74**:7422-7426) to form pPS3559 where *folE* transcription is driven by the arabinose-inducible BAD promoter (P_{BAD}). The pHERD20T and pPS3559 plasmids were transformed into *E. coli* strain MG1655 Δ*folE*::Km^r, a kind gift of Dr. Valérie de Crécy-Lagard, University of Florida (**El Yacoubi B** et al. 2006. J Biol Chem **49**:37586-37593). Transformants were selected on LB medium supplemented with 100 μg/ml ampicillin (Ap) and 0.3 mM thymidine (Thy) to allow slow growth of the Δ*folE* host strain. To assess complementation with *B. pseudomallei folE*, transformants with empty vector, vector with *folE*, and the untransformed Δ*folE* host strain were streaked on LB medium with 0.3 mM Thy (top left panel in the figure), 0.3 mM Thy + Ap (top right panel), Ap (bottom left panel), and Ap + 0.01% arabinose. The plates were incubated at 37°C for 48 h. The numbers indicate: 1, MG1655 Δ*folE*::Km^r; 2, MG1655 Δ*folE*::Km^r transformed with vector pHERD20T; 3 & 4, MG1655 Δ*folE*::Km^r transformed with pPS3559 (pHERD20T with *folE* under transcriptional control of P_{BAD}). As expected, the *E. coli* Δ*folE* strain exhibits a slow growth phenotype on LB + Thy medium (top left panel) and does not grow on LB + Thy + Ap medium (top right panel). The Thy auxotrophy is relieved by pPS3559 but not the pHERD20T vector control (bottom panels). While complementation is observed in the absence of arabinose induction (probably due to leaky expression from P_{BAD}) (bottom left panel), growth is more robust on the plate containing 0.01% arabinose (bottom right panel).
