# Supplementary material for: Mechanisms of Resistance to Folate Pathway Inhibitors in Burkholderia pseudomallei: Deviation from the Norm
Source: mBio. 2017 Sep 5;8(5):e01357-17. doi: 10.1128/mBio.01357-17 (PMC5587915; doi:10.1128/mBio.01357-17)
Supplement: TABLE S1 [file mbo004173469st1.pdf]

Table S1. ***Burkholderia pseudomallei*** **strains used in this study**

Strain	Description and relevant genotypes[1]	Reference
Bp82	*B. pseudomallei* attenuated and select agent excluded prototype strain	(1)
**Bp82-derived TMP^r selected mutants**		
Bp82.102	Bp82 $bpeT_{L265R}$	This study
Bp82.103	Bp82 $bpeT_{C310R}$ $folA_{F158V}$	This study
Bp82.104	Bp82 $folA_{I99L}$	This study
**Bp82-derived TMP^r reconstructed mutants**		
Bp82.183	Bp82 $folA_{F158V}$	This study
Bp82.184	Bp82 $folA_{I99L}$	This study
Bp82.268	Bp82 $bpeT_{C310R}$	This study
Bp82.269	Bp82 $bpeT_{L265R}$	This study
**Bp82-derived SXT^r selected mutants**		
Bp82.191	Bp82 $bpeS_{K267T}$ $folM_{V15G}$	This study
Bp82.193	Bp82 $bpeS_{K267T}$ $folM_{\Delta A203}$	This study
Bp82.199	Bp82 $bpeS_{K267T}$ $folM_{V15G}$	This study
Bp82.202	Bp82 $bpeS_{K267T}$ $folM_{V15G}$	This study
Bp.82.204	Bp82 $bpeS_{K267T}$ $folM_{V15G}$	This study
Bp82.207	Bp82 $bpeS_{K267T}$ $folM_{V15G}$	This study
**Repair of $folM_{V15G}$ and $bpeS_{K267T}$ SNPs in SXT^r isolates**		
Bp82.246	Bp82.202 $folM_{V15G}$ $bpeS_{WT}$	This study
Bp82.247	Bp82.202 $folM_{WT}$ $bpeS_{K267T}$	This study
Bp82.248	Bp82.202 $folM_{WT}$ $bpeS_{WT}$	This study
Bp82.249	Bp82.204 $folM_{V15G}$ $bpeS_{WT}$	This study
Bp82.250	Bp82.204 $folM_{WT}$ $bpeS_{K267T}$	This study
Bp82.251	Bp82.204 $folM_{WT}$ $bpeS_{WT}$	This study
**Bp82 with *bpeS* point mutations**		
Bp82.284	$bpeS_{P29S}$	This study
Bp82.285	$bpeS_{K267T}$	This study
**Deletion of *folM*, *bpeT* and *bpeS* in Bp82**		
Bp82.262	Bp82 Δ*folM*	This study
Bp82.264	Bp82 Δ*bpeS*	This study
Bp82.286	Bp82.264 $\Delta bpeT_{572}$[2]	This study
**Constructed efflux mutants**		
Bp82.253	Bp82 $\Delta bpeT_{1008}$[2]	This study
Bp82.27	Bp82 Δ(*amrAB-oprA)*	This study
Bp82.57	Bp82.27 Δ*(bpeAB-oprB*)	This study
Bp82.87	Bp82.57 $\Delta bpeT_{572}$[2]	This study

Table S1 (Continued)

Strain	Description and relevant genotypes[1]	Reference
**Bp82 BpeT and BpeS overexpressing strains**		
Bp82.187	Bp82.87::mini-Tn*7*T-Gm-*P1-bpeT*	This study
Bp82.189	Bp82.87::mini-Tn*7*T-Gm	This Study
Bp82.323	Bp82.264::mini-Tn*7*T-Km	This Study
Bp82.324	Bp82.286::mini-Tn*7*T-Km	This Study
Bp82.288	Bp82.286::mini-Tn*7*T-Km-*P1-bpeS*	This Study
Bp82.289	Bp82.264::mini-Tn*7*T-Km-*P1-bpeS*	This Study
Bp82.310	Bp82.264::mini-Tn*7*T-Km-*P1-bpeS*$_{\mathrm{P29S}}$	This study
Bp82.311	Bp82.286::mini-Tn*7*T-Km-*P1-bpeS*$_{\mathrm{P29S}}$	This study
Bp82.320	Bp82.264::mini-Tn*7*T-Km-*P1-bpeS*$_{\mathrm{K267T}}$	This Study
Bp82.321	Bp82.286::mini-Tn*7*T-Km-*P1-bpeS*$_{\mathrm{K267T}}$	This Study
**SXT resistant clinical isolates**		
MSHR663	Relapse isolate of MSHR664, Australia, October 1998	(2)
MSHR664	Pneumonia blood culture, Australia, March 1998	Menzies Collection
MSHR8441	Cystic fibrosis isolate, Australia, 2010	(3)
MSHR8442	Cystic fibrosis isolate, Australia, isolated 14 months after MSHR8441	(3)
354b	Sputum isolate, Thailand, 1988	(4)
354e	Relapse sputum isolate, Thailand, 1994	(4)
5041a	Sputum isolate, Ubon Thailand, 2008	Mahidol Collection
1374a	Pus isolate, Ubon Thailand, 1995	(5, 6)
Bp1651	Cystic fibrosis isolate, USA	(7)
**Antibiotic susceptible clinical isolate**		
1026b	Blood isolate, Thailand, 1993	(4)

[1]**Abbreviations:** *FRT*, Flp recombinase target site; Gm, gentamicin resistance determinant; Km, kanamycin resistance determinant; ^{r}, resistant; SNP, single nucleotide polymorphism; SXT, co-trimoxazole (trimethoprim:sulfamethoxazole, 1:19); TMP, trimethoprim; WT, wild-type Bp82 sequence.
[2]Suffixes 572 and 1008 indicate an internal 572 bp *bpeT* deletion and a 1,008 bp complete *bpeT* deletion, respectively.

References

1. **Propst KL, Mima T, Choi KH, Dow SW, Schweizer HP.** 2010. A *Burkholderia pseudomallei* Δ*purM* mutant is avirulent in immune competent and immune deficient animals: candidate strain for exclusion from Select Agent lists. Infect Immun **78:**3136-3143.
2. **Sarovich DS, Ward L, Price EP, Mayo M, Pitman MC, Baird RW, Currie BJ.** 2014. Recurrent melioidosis in the Darwin Prospective Melioidosis Study: improving therapies mean that relapse cases are now rare. J Clin Microbiol **52:**650-653.

3. **Viberg LT, Sarovich DS, Kidd TJ, Geake JB, Bell SC, Currie BJ, Price EP.** 2017. Within-host evolution of *Burkholderia pseudomallei* during chronic infection of seven Australasian cystic fibrosis patients. mBio **8**:e00356-17. DOI:10.1128/mBio.00356-17.
4. **Hayden HS, Lim R, Brittnacher MJ, Sims EH, Ramage ER, Fong C, Wu Z, Crist E, Chang J, Zhou Y, Radey M, Rohmer L, Haugen E, Gillett W, Wuthiekanun V, Peacock SJ, Kaul R, Miller SI, Manoil C, Jacobs MA.** 2012. Evolution of *Burkholderia pseudomallei* in recurrent melioidosis. PLoS One **7:**e36507. DOI:10.1371/journal.pone.0036507.
5. **Dance DA, Davong V, Soeng S, Phetsouvanh R, Newton PN, Turner P.** 2014. Trimethoprim/sulfamethoxazole resistance in *Burkholderia pseudomallei*. Int J Antimicrob Agents **44:**368-369.
6. **Saiprom N, Amornchai P, Wuthiekanun V, Day NP, Limmathurotsakul D, Peacock SJ, Chantratita N.** 2015. Trimethoprim/sulfamethoxazole resistance in clinical isolates of *Burkholderia pseudomallei* from Thailand. Int J Antimicrob Agents **45:**557-559.
7. **Bugrysheva JV, Sue D, Gee JE, Elrod MG, Hoffmaster AR, Randall LB, Chirakul S, Tuanyok A, Schweizer HP, Weigel LM.** 2017. Antibiotic resistance markers in strain Bp1651 of *Burkholderia pseudomallei* Identified by genome sequence analysis. Antimicrob Agents Chemother. **61**:e00010-17. DOI:10.1128/AAC.00010-17.
