# Supplementary material for: Mechanisms of Resistance to Folate Pathway Inhibitors in Burkholderia pseudomallei: Deviation from the Norm
Source: mBio. 2017 Sep 5;8(5):e01357-17. doi: 10.1128/mBio.01357-17 (PMC5587915; doi:10.1128/mBio.01357-17)
Supplement: TABLE S2 [file mbo004173469st2.pdf]

Table S2. Antimicrobial susceptibilities resulting from BpeT and BpeS overexpression

Strain and Relevant Genotype[1]	Minimum Inhibitory Concentration (µg/ml) TMP[2]	SMX[2]	SXT
Bp82	0.75	4	0.094
Bp82.189 Δ*bpeT*[3]	1	8	0.094
Bp82.187 Δ*bpeT P1-bpeT*	16	128	1
Bp82.289 Δ*bpeS P1-bpeS*$_{\mathrm{WT}}$	0.75	6	0.094
Bp82.288 Δ*bpeS* Δ*bpeT P1-bpeS*$_{\mathrm{WT}}$	0.38	4	0.125
Bp82.320 Δ*bpeS P1-bpeS*$_{\mathrm{K267T}}$	≥ 32	≥ 1024	6
Bp82.321 Δ*bpeS* Δ*bpeT P1-bpeS*$_{\mathrm{K267T}}$	≥ 32	≥ 1024	6
Bp82.310 Δ*bpeS P1- bpeS*$_{\mathrm{P29S}}$	≥ 32	≥ 1024	6
Bp82.311 Δ*bpeS* Δ*bpeT P1-bpeS*$_{\mathrm{P29S}}$	≥ 32	≥ 1024	6

[1]All strains are based on Bp82 except Bp82.187 and Bp82.189, which are derived from Δ(*amrAB-oprA*) Δ(*bpeAB-oprB*)strain Bp82.87 (**Supplementary Table S1**). Bp82.87 was employed to enable selection for the gentamicin resistance marker contained of the mini-Tn*7* element used for chromosomal integration of the *P1-bpeT* construct.

[2]The detection limit for the Etest® assay is 32 µg/ml for Tmp and 1024 µg/ml for SMX.

[3]Empty vector control.
