# Supplementary material for: Mechanisms of Resistance to Folate Pathway Inhibitors in Burkholderia pseudomallei: Deviation from the Norm
Source: mBio. 2017 Sep 5;8(5):e01357-17. doi: 10.1128/mBio.01357-17 (PMC5587915; doi:10.1128/mBio.01357-17)
Supplement: TABLE S3 [file mbo004173469st3.pdf]

**Table S3. The BpeEF-OprC efflux pump is required for increased folate inhibitor resistance in laboratory strains and clinical isolates.**

| | Minimal Inhibitory Concentration (μg/ml) | | | | | | | | | | | |
|---|---|---|---|---|---|---|---|---|---|---|---|---|
| | *bpeE*[+]*F*[+]*-oprC*[+] | | | Δ(*bpeEF-oprC*) | | | Δ(*bpeEF-oprC*)::mini-Tn*7*T-$P_{tac}$-*bpeEF-oprC* | | | Δ(*bpeEF-oprC*)::mini-Tn*7*T-$P_{tac}$-*bpeEF-oprC* + 1 mM IPTG[2] | | |
| Strain | TMP[1] | SMX[1] | SXT | TMP | SMX | SXT | TMP | SMX | SXT | TMP | SMX | SXT |
| Bp82.191 | ≥ 32 | ≥ 1024 | 4 | 1.5 | 8 | 0.125 | 1.5 | 12 | 0.125 | 12 | 16 | 0.5 |
| Bp82.193 | ≥ 32 | ≥ 1024 | 3 | 1.5 | 6 | 0.094 | 1 | 6 | 0.094 | 6 | 8 | 0.5 |
| Bp82.199 | ≥ 32 | ≥ 1024 | 2 | 1.5 | 6 | 0.125 | 1.5 | 12 | 0.125 | 8 | 24 | 0.75 |
| Bp82.202 | ≥ 32 | ≥ 1024 | 6 | 1.5 | 6 | 0.125 | 1.5 | 16 | 0.125 | 12 | 24 | 0.5 |
| Bp82.204 | ≥ 32 | ≥ 1024 | 4 | 2 | 8 | 0.125 | 1.5 | 12 | 0.125 | 6 | 16 | 0.5 |
| Bp82.207 | ≥ 32 | ≥ 1024 | 2 | 1.5 | 12 | 0.125 | 1.5 | 16 | 0.125 | 8 | 24 | 0.5 |
| 1374a | ≥ 32 | ≥ 1024 | 4 | 1.5 | 8 | 0.125 | - | - | - | - | - | - |
| 5041a | ≥ 32 | ≥ 1024 | 4 | 16 | 16 | 0.19 | - | - | - | - | - | - |

[1] The detection limit for the Etest® assay is 32 μg/ml for TMP and 1024 μg/ml for SMX.
[2]IPTG, isopropyl-β-D-thiogalactopyranoside.
