# Supplementary material for: Mechanisms of Resistance to Folate Pathway Inhibitors in Burkholderia pseudomallei: Deviation from the Norm
Source: mBio. 2017 Sep 5;8(5):e01357-17. doi: 10.1128/mBio.01357-17 (PMC5587915; doi:10.1128/mBio.01357-17)
Supplement: FIG S1 [file mbo004173469sf1.pdf]

Figure S1. FolM gene and protein mutations in laboratory selected Bp82 and clinical SXT resistant isolates.

A)

```
 40 CTCGTGACGGGCGCCGCGCGCCGCGCGGGCCGCG             1026b, Bp82
 40 CTCGgGACGGGCGCCGCGCGCCGCGCGGGCCGCG             Bp82.202, Bp82.204
 40 CTCGTGACGG-------GCGCCGCGCGGGCCGCG             MSHR663, 354e, 5041a
 40 CTCGTGACGGGCGCcgcgcgccgCGCGCGCCGCGCGGGCCGCG    MSHR8441

200 CGGACCTTGCGCACGCGGATGCGATCAC                   1026b, Bp82
200 CGG-CCTTGCGCACGCGGATGCGATCAC                   Bp82.193
```

B)

```
   C     C
   IVL   S    F         Y
   VII   G   RL         F      NADPH binding site
   ALVTGAXXGIGXXXXXXL          consensus
11 PVVLVTGAARRAGRAFALRFA       1026b, Bp82
11 PVVLGTGAARRAGRAFALRFA       Bp82.202, Bp82.204
11 PVVLVTGAARAARSRCASPRA       MSHR663, 354e, 5041a
11 PVVLVTGAARRARRAGRAFALRFA    MSHR8441, MSHR8442

60 GEAVALQADLAHADAITQLVD       1026b, Bp82
60 GEAVALQAALRTRMRSRSSSM       Bp82.193
```

A) Mutations in *folM* affecting the integrity of the 267 amino acid FolM protein. Point, deletion and insertion mutations indicated by a single red letter, dashes and letters, respectively. **B**) Amino acid changes (red letters) caused by the *folM* mutations shown in panel **A**. The top line in panel **A** shows the wild-type *folM* sequences found in 1026b and its select agent excluded derivative Bp82. Bp82.202 and Bp82.204 are laboratory selected SXT^{r} derivatives of Bp82 with a T to G change at nucleotide 44 that causes a threonine to glycine change at position 15 of FolM. Bp82.193 is laboratory selected SXT^{r} derivative of Bp82 with a single nucleotide deletion at position 203 in *folM* that causes a frame shift after amino acid 67 of FolM. Strains MSHR663, 354e and 5041a are clinical isolates with identical 7 bp deletions after nucleotide 49 of *folM*. In all three strains, the 7 bp insertion leads to an identical frame shift after amino acid 20 of FolM and synthesis of a prematurely terminated 90 amino acid protein. Lastly, clinical isolates MSHR8441 and MSHR8442 contain a 9 bp nucleotide insertion after *folM* nucleotide 53. This results in a duplication of amino acids 20-22 and synthesis of a 270 amino acid FolM protein. In all instances but one (Bp82.193) the mutations affect the integrity of the predicted NADPH-binding site consensus indicated in the green box at the top. The white letters in the black boxes indicate invariant T and G NADPH-binding site residues (**Hua** et al. 2014. Sci Rep **4**:doi:10.1038/srep06471).
