# Supplementary material for: Mechanisms of Resistance to Folate Pathway Inhibitors in Burkholderia pseudomallei: Deviation from the Norm
Source: mBio. 2017 Sep 5;8(5):e01357-17. doi: 10.1128/mBio.01357-17 (PMC5587915; doi:10.1128/mBio.01357-17)
Supplement: FIG S2 [file mbo004173469sf2.pdf]

**Figure S2. Clinical and laboratory strains containing chromosomal *bpeS*$_{P29S}$ express high-levels of *bpeF*.**

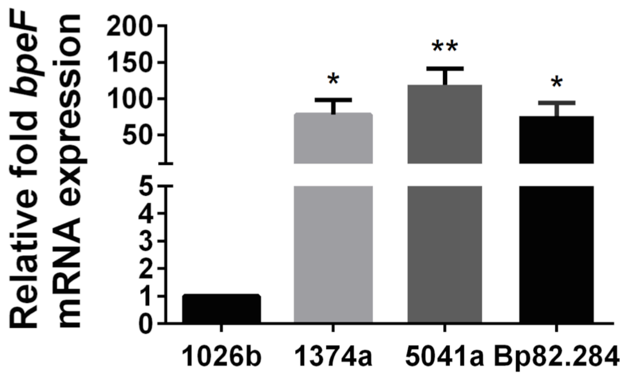


Total RNA was isolated from cells of the indicated strains grown to mid-log phase in LB medium. The expression of *bpeF* was measured by RT-qPCR and one-way ANOVA was performed with Dunnet's post-test to determine statistical significance compared to the 1026b control. Bars represent mean expression of at least two biological replicates with one standard deviation. The data show that presence of *bpeS*$_{P29S}$ allele caused *bpeF* over-expression. (**, $p < 0.01$; *, $p \leq 0.05$).
