# Supplementary material for: Mechanisms of Resistance to Folate Pathway Inhibitors in Burkholderia pseudomallei: Deviation from the Norm
Source: mBio. 2017 Sep 5;8(5):e01357-17. doi: 10.1128/mBio.01357-17 (PMC5587915; doi:10.1128/mBio.01357-17)
Supplement: FIG S3 [file mbo004173469sf3.pdf]

Figure S3. BpeS and BpeT are dispensable for expression of *bpeEF-oprC* in the presence of some pump substrates

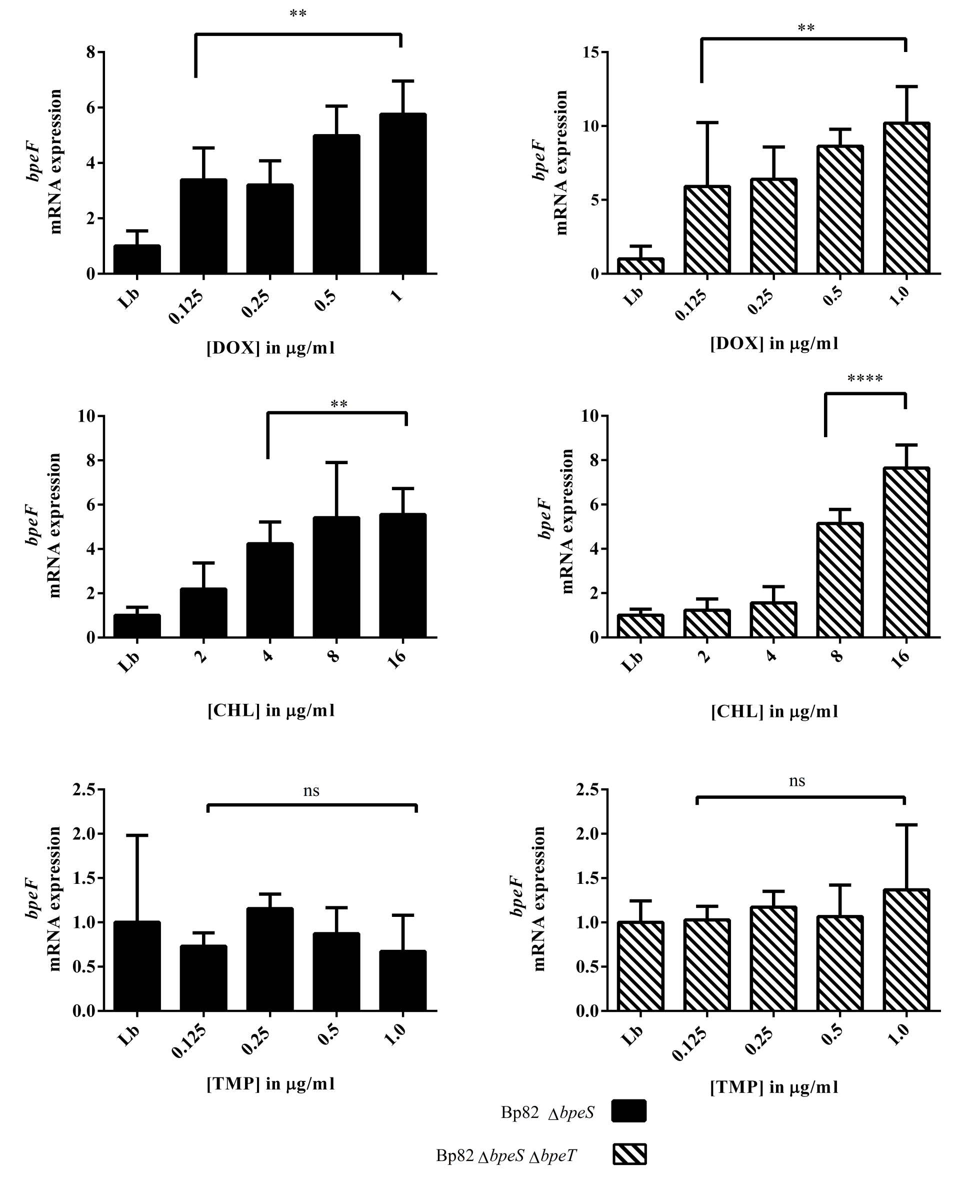

Bp82 and its derivatives Bp82 Δ*bpeS* (Bp82.264) or Bp82 Δ*bpeS* Δ*bpeT* (Bp82.286) were grown overnight in LB medium supplemented with 80 mg/ml adenine, subcultured into the same medium (1:50 dilution), and incubated with shaking at 37°C until the culture reached mid-log phase (OD_{600nm} = 0.6-0.8). The culture was split into four aliquots. The control culture received no antibiotic. To the other three cultures either trimethoprim (TMP), or doxycycline (DOX) or chloramphenicol (CHL), was added at concentrations ranging from 2 to 16 µg/ml in 2-fold increments. The cultures were incubated with shaking at 37°C for one hour before total RNA was extracted. RNA was extracted, converted to cDNA and RT-qPCR as previously described (**Podnecky NL** et al. 2013. Antimicrob Agents Chemother **57**:4381-4386). RT-qPCR analysis was performed to determine *bpeF* expression at each drug concentration. Bars depict mean *bpeF* expression fold change with error bars indicating one standard deviation. All samples are compared to the untreated LB control in two biological replicates. Two-way ANOVA and Dunnet's multiple comparison were performed in GraphPad Prism to determine significant differences from the untreated LB control. With the exception of TMP tests, significant *bpeF* expression occurred in all other samples tested. ****, $p < 0.0001$, **, $p < 0.01$.

The data confirm our previous observations that *bpeEF-oprC* expression is inducible in response to select BpeEF-OprC pump substrates. In Bp82 and Bp82Δ*bpeT*, relative *bpeF* mRNA levels varied in a dose-dependent manner after 1-hour incubation in the presence of increasing concentrations of CHL or DOX (data not shown). In strains lacking only *bpeS* (Bp82.264), we observed a similar result with dose-dependent increases in *bpeF* expression detected in response to CHL or DOX exposure. Interestingly, in the absence of both *bpeS* and *bpeT (*Bp82.286), expression levels of *bpeF* were significantly increased in response to the presence of DOX or CHL, as was observed in the wild-type Bp82, Δ*bpeT,* and Δ*bpeS* strains. Wild-type like induction occurs in the absence of both genes, indicating neither BpeT nor BpeS appears to be solely responsible for expression of *bpeEF-oprC*. In contrast, these same isolates were unable to promote expression of *bpeF* with 1-hour exposure to TMP. These data support the notions 1) that an additional regulatory factor is responsible for CHL and DOX related pump induction, and 2) that the co-inducer specificities of BpeS, BpeT and perhaps any additional factors may vary to enable highly adaptable control of *bpeEF-oprC* expression.
