# Supplementary material for: Mechanisms of Resistance to Folate Pathway Inhibitors in Burkholderia pseudomallei: Deviation from the Norm
Source: mBio. 2017 Sep 5;8(5):e01357-17. doi: 10.1128/mBio.01357-17 (PMC5587915; doi:10.1128/mBio.01357-17)
Supplement: TEXT S1 [file mbo004173469s1.pdf]

Supplemental Methods

General methods

All chromosomal deletion and allelic exchange procedures in *B. pseudomallei* strain Bp82 (bacterial strains used in this study are listed in **Table S1**) were performed using pEXKm5 (1) or pEXGm5B (see **Table S5** for plasmids used in this study). The gene/allele replacement vector pEXGm5B was derived from pEXKm5 by replacing a *Spe*I-*Xba*I fragment containing the kanamycin resistance with a gentamicin resistance marker located on a *Xba*I fragment from pFGM1 (2). Merodiploids were obtained by transforming the allele replacement constructs into *E. coli* mobilizer strain RHO3 (1). After conjugation with the desired Bp82 derivative, merodiploid transformants were selected on low salt (5 g/l NaCl) LB medium containing kanamycin (pEXKm5) or gentamicin (pEXGm5B) supplemented with 80 µg/ml adenine for strain Bp82 and its derivatives. The concentrations of antibiotics used were 1,000 µg/ml kanamycin for AmrAB-OprA expressing strains and 35 µg/ml kanamycin for Δ(*amrAB-oprA*) strains or 2,000 µg/ml gentamicin for AmrAB-OprA expressing strains and 15 µg/ml gentamicin for Δ(*amrAB-oprA*) strains. Merodiploid resolution was performed using previously described protocols, using either sucrose or I-*Sce*I for promotion of excision of unwanted DNA sequences (1, 2). Resistance markers flanked by Flp recombinase target (*FRT*) sequences were excised using pFLPe2 or pFLPe4 as previously described (2). The presence of the desired mutations in mutants was confirmed by PCR amplification of the mutation-containing region and DNA sequencing of the amplicon. Sequence verification of inserts cloned into pGEM-T Easy (Promega, Madison, WI) or pCR2.1 (Thermofisher, Waltham, MA) was achieved using primers M13F-20 (5'-GTAAAACGACGGCCAGT) and M13R (5'-AACAGCTATGA-CCATG) (3). Unless noted otherwise, Bp82 genomic DNA was used for PCR amplification of DNA fragments used in mutant construction. PCR fragments were purified from agarose gels using the GenElute DNA Extraction kit (Sigma Aldrich, St. Louis, MO).

Isolation of deletion mutants in Bp82

Deletion of an internal DNA fragment from *bpeT*. A Δ*bpeT* construct was created by first amplifying the *bpeT* gene using Platinum Taq DNA polymerase High Fidelity and primers 1790 + 1791 (PCR primers and mutagenic oligonucleotides are listed in **Table S4**) and ligating the resulting fragment into pCR2.1 to create pPS2565. Second, a 834-bp *FRT-ble-FRT* zeocin resistance cassette obtained from pFZE1 (2) was released by *Sal*I digestion (unless indicated otherwise, restriction enzymes were obtained from New England BioLabs) and then inserted between the *Sal*I sites of the *bpeT* gene. This procedure deleted 572 bp from *bpeT* (nucleotides 292-863 of the 1,004 nucleotide *bpeT* coding sequence) and created pPS2567. This plasmid was then digested with *Eco*RI and Δ*bpeT*::*FRT-ble-FRT* fragment ligated into the *Eco*RI site of pEXKm5 to yield pPS2571. Finally, pPS2647 was obtained by *Sal*I digestion and religation to delete the *FRT-ble-FRT* cassette. Chromosomal 572-bp *bpeT* deletions obtained with pPS3144 were verified by PCR amplification with primers 1790 + 1791 and sequencing of the amplicon.

Complete deletion of *bpeT*. A second *bpeT* deletion construct was built by assembling PCR products obtained with Platinum Taq polymerase High Fidelity and primers 2636 + 2643 and 2638 + 2637 using splicing by overlap extension (SOEing) PCR. The resulting 1,018 bp PCR fragment contained a complete 1,008 bp *bpeT* deletion from start to stop codon and flanking DNA for recombination. This fragment was

ligated into pGEM-T Easy, which was subsequently digested with *Not*I and a 1,052 bp fragment containing the Δ*bpeT* allele with flanking sequences for recombination was ligated into the pEXKm5 to generate pPS3137. Chromosomal 1,008-bp *bpeT* deletions obtained with pPS3137 were verified by PCR amplification with primers 2636 + 2637 to show fragment loss.

Deletion of *bpeS*. For deletion of the *bpeS* gene, primer pairs 2618 + 2619 and 2622 + 2623 and Platinum Taq DNA polymerase High Fidelity were used to PCR amplify 606 bp and 664 bp DNA fragments, respectively, upstream and downstream of the *bpeS* gene from genomic DNA. These products were assembled with a 1,420-bp fragment containing the *FRT-nptII*-FRT kanamycin resistance marker amplified with primers 2620 + 2621 from pFKM2 (2) using SOEing PCR and primers 2619 + 2622. The 2,641-bp SOEing PCR product was sequentially cloned into pGEM-T Easy (yielding pPS3127) and then as a *Not*I fragment into the *Not*I site of pEXGm5B to obtain pPS3158. Chromosomal *bpeS* deletion strains obtained with pPS3158 have 1,321 bp of the *bpeS* region deleted (its entire coding sequence plus 217 bp upstream and 132 bp downstream sequence). The presence of the Δ*bpeS* allele in these mutants was verified by PCR amplification with primers 2618 + 2623 to show fragment loss.

Deletion of *folM*. To build the deletion construct for the *folM* gene, the PCR products obtained with Platinum Taq DNA polymerase High Fidelity and primers 2639 + 2640 and 2641 + 2642 were assembled by SOEing PCR and the resulting 1,236-bp fragment was cloned into pGEM-T Easy vector to form pPS3130. This plasmid was digested with *Hin*dIII, which cut at a site introduced in the SOEing primers between the flanking DNA fragments. A *FRT-nptII-FRT* cassette was excised on a 1,514-bp *Hin*dIII fragment from pFKM2 and was ligated into the *Hin*dIII site located between the cloned chromosomal DNA fragments in pPS3130, generating pPS3140. The *folM* deletion construct was excised from pPS3140 with *Not*I and cloned into the same site of pEXGm5B to yield pPS3144. Chromosomal *folM* deletion strains obtained with pPS3144 have 788 bp of the 801 bp *folM* coding sequence deleted. The presence of the Δ*folM* allele in these strains was verified by PCR amplification with primers 2640 + 2641 to show fragment loss.

Deletion of efflux pump operons. Deletion of the *amrAB-oprA, bpeAB-oprB,* and *bpeEF-oprC* genes was achieved using pPS2833, pPS2899, and pPS2591 (4), respectively. The gene replacement vector pPS2833 was derived in several steps. First, a 4,451 *Sac*I fragment was deleted from the *amrAB-oprA* operon located on pPS2142 (2) and the plasmid backbone containing the *amrAB-oprA* deletion was religated. Second, an *Eco*RI fragment containing Δ(*amrAB-oprA*) was excised from the resulting plasmid and cloned into pEXKm5 to form pPS2833. For construction of pPS2899, an *Eco*RI fragment containing Δ(*bpeAB-oprB)-FRT-Km-FRT* from pPS2412 (5) was cloned into the same site of pEXGm5B.

Construction of mutants containing chromosomal point mutations

Chromosomal *folA* point mutations. For construction of mutants containing *folA* alleles, Platinum Taq DNA polymerase High Fidelity and primers 1966 + 1967 were used to PCR amplify the *folA* gene and flanking regions from both Bp82.102 ($folA_{F158V}$) and Bp82.104 ($folA_{I99L}$) genomic DNA. The 694-bp PCR products were initially cloned into pGEM-T Easy to create pPS2951 ($folA_{F158V}$) and pPS2552 ($folA_{I99L}$), respectively. After confirmation of the correct insert, DNA fragments containing the *folA* mutations were excised by *Eco*RI and ligated into the same site of pEXKm5, resulting in pPS2959 ($folA_{F158V}$) and

pPS2960 (*folA*$_{I99L}$), respectively. These plasmids were used to introduce the *folA*$_{F158V}$ and *folA*$_{I99L}$ alleles into Bp82 to create Bp82.183 and Bp82.184, respectively.

Chromosomal *bpeT* point mutations. Phusion High Fidelity PCR Master Mix (New England BioLabs) and primers 2636 + 2637 were used to amplify the *bpeT* gene and flanking regions from genomic DNA of both Bp82.102 (*bpeT*$_{C310R}$) and Bp82.103 (*bpeT*$_{L265R}$). The 2,026-bp PCR products were purified from an agarose gel and after addition of single 3' adenine base overhangs with Taq polymerase (New England BioLabs) ligated into pGEM-T Easy to create pPS3167 (*bpeT*$_{C310R}$) and pPS3168 (*bpeT*$_{L265R}$), respectively. *Pvu*II fragments containing the correct mutant *bpeT* alleles were ligated into *Sma*I linearized pEXKm5, resulting in pPS3177 (*bpeT*$_{C310R}$) and pPS3178 (*bpeT*$_{L265R}$). These plasmids were used to introduce the *bpeT*$_{C310R}$ and *bpeT*$_{L265R}$ alleles into Bp82 to create Bp82.268 and Bp82.269.

Repair of genomic single nucleotide polymorphisms (SNPs) by allelic replacement

Repair of *folM*$_{V15G}$. Platinum Taq DNA polymerase High Fidelity and primers 2640 + 2641 were used to PCR amplify the *folM* gene and flanking regions from genomic DNA of Bp82 (wild-type for *folM*). The 1,560-bp PCR product was cloned into pGEM-T Easy to yield pPS3099. The insert was then excised on a *Not*I fragment and ligated into the same site of pEXKm5 plasmid to yield pPS3093. This plasmid was used to repair *folM*$_{V15G}$ in strains Bp82.202 and Bp82.204.

Repair of *bpeS*$_{K267T}$. Platinum Taq DNA polymerase High Fidelity and primers 2618 + 2623 were used to amplify the *bpeS* gene from genomic DNA of Bp82 (wild-type for *bpeS*). The resulting 1,456-bp PCR fragment was cloned into pGEM-T Easy to form pPS3097. The *bpeS*-containing insert was excised from pPS3097 by digestion with *Not*I and the resulting fragment was ligated into the *Not*I site of pEXKm5 to yield pPS3090. This plasmid was used to repair *bpeS*$_{K267T}$ in strains Bp82.202 and Bp82.204.

Constructs for introduction of *bpeS*$_{K267T}$ into the chromosome. A1,456-bp fragment containing *bpeS*$_{K267T}$ was PCR amplified from Bp82.204 genomic DNA using Platinum Taq DNA polymerase High Fidelity with primers 2570 + 2573 and cloned into pGEM-T Easy to create pPS3098. Next, 2,542 bp region containing *bpeS* and homologous sequence was PCR amplified from Bp82 genomic DNA primers 2618 + 2623. The resulting DNA fragment was cloned into pGEM-T Easy to create pPS3180. Finally, a 1,204 bp *Eco*RV + *Xma*I fragment from pPS3180 was replaced with the same fragment from pPS3098, which produced pPS3187. The *bpeS*$_{K267T}$ containing *bpeS* fragment was recovered from pPS3187 by *Not*I digestion and ligated into the *Not*I site of pEXKm5 to create pPS3190.

Constructs for introduction of *bpeS*$_{P29S}$ into the chromosome. First, a 1,456-bp fragment containing the wild-type *bpeS* was PCR amplified from Bp82 genomic DNA using Platinum Taq DNA polymerase High Fidelity with primers 2570 + 2573 and cloned into pGEM-T Easy to create pPS3097. Second, the *bpeS*$_{P29S}$ construct was built by introducing the SNP mutation into the wild- *bpeS* containing construct (pPS3097) using the QuikChange II XL Site Directed Mutagenesis kit (Agilent Technologies, Santa Clara, CA) with primers 2607 + 2608, following the manufacturer's guidelines. The resulting pPS3106 was confirmed by DNA sequencing with M13F-20 + M13R. Third, a 1,204 bp *Eco*RV + *Xma*I fragment from pPS3180 was replaced with the same fragment from pPS3106, which produced pPS3186. Fourth,

the *bpeS*$_{P29S}$ containing fragment was recovered from pPS3186 by *Not*I digestion and ligated into the *Not*I site of pEXKm5 to create pPS3189.

Construction of *bpeT* and *bpeS* expressing mini-Tn*7* elements

Mini-Tn*7-P1-bpeT* expression vectors. The *P1* promoter was PCR amplified from p34E-Tp1 (6) using Platinum Taq DNA polymerase High Fidelity and primers 1563 + 1789 on a 399 bp DNA fragment. A 1,201 bp promoter-less *bpeT* DNA fragment was PCR amplified from 1026b genomic DNA with primers 1790 + 1791. These two DNA fragments were assembled by SOEing PCR using primers 1563 + 1791 to create a 1,600 bp fragment. This fragment was cloned into pGEM-T Easy to create pPS2453. The insert was recovered from this plasmid on an *Nsi*I-*Nru*I fragment and inserted between the same sites of pUC18T-mini-Tn*7*T-Gm-*FRT* (7) to create pPS2463. The mini-Tn*7-P1-bpeT* element was transferred into the Δ*bpeT* strain Bp82.87 by co-electroporation of pPS2463 and pTNS3 as described elsewhere (2). An empty mini-Tn*7*T-Gm vector was similarly transferred as a control. Strains containing either a PCR confirmed mini-Tn*7*T-Gm-*P1-bpeT* (Bp82.187) or mini-Tn*7*T-Gm (Bp82.189) insertion at the *glmS1* associated *att*Tn*7* site were retained. Bp82.87 was employed as the host strain to enable selection for the gentamicin resistance marker contained of the mini-Tn*7* element used for chromosomal integration of the *P1-bpeT* construct.

Mini-Tn*7-P1-bpeS* expression vectors. To create a mini-Tn*7-P1-bpeS* construct, Platinum Taq DNA polymerase High Fidelity and primers 2702, 2693, 2694 and P2696 were utilized to PCR amplify the wild-type *bpeS* gene from plasmid pPS3097, as well as recover the *P1* promoter containing fragment from plasmid pTNS3 (2). The *P1* promoter region and *bpeS* amplicons were assembled by SOEing PCR using primers 2693 + 2702 to produce a 1,450 bp DNA fragment. This fragment was cloned into pGEM-T Easy to create pPS3196 and confirmed by sequencing. The 1,450 bp fragment was excised from pPS3196 using *Kpn*I+*Hin*dIII and ligated between the same sites of pUC18T-mini-Tn*7*T-Km (2) to create pPS3198. The plasmid was confirmed using *Kpn*I+*EcoR*I digest and sequencing. Plasmids pPS3198 or pUC18T-mini-Tn*7*T-Km were electroporated into Δ*bpeS* strain Bp82.264 and Δ*bpeS* Δ*bpeT* strain Bp82.286 along with the pTNS3 helper plasmid. Strains containing either a PCR confirmed mini-Tn*7*T-Km (Bp82.323 and Bp82.324) or mini-Tn*7*T-Km-*P1-bpeS* (Bp82.288 and Bp82.289) insertion at the *glmS3* associated *att*Tn*7* site were retained.

A mini-Tn*7-P1-bpeS*$_{K267T}$ expression vector was constructed by PCR amplification of a 979 bp *bpeS*$_{K267T}$ containing fragment from pPS3190 DNA using Platinum Taq DNA polymerase High Fidelity and primers 2693 + 2871. Cloning of this fragment into pGEM-T Easy created pPS3262. A 946 bp *Ale*I-*Kpn*I fragment containing *bpeS*$_{K267T}$ was recovered from pPS3262 and used to replace the resident *Ale*I-*Kpn*I fragment of pUC18T-mini-Tn*7*T-Km-*P1-bpeS* (or pPS3198) to create pUC18T-mini-Tn*7*T-Km-*P1-bpeS*$_{K267T}$ (or pPS3280). The mini-Tn*7*-Km-*P1-bpeS*$_{K267T}$ containing construct pPS3280 and pTNS3 were co-electroporated into Bp82.264 and Bp82.286. Strains Bp82.320 and Bp82.321containing a PCR confirmed mini-Tn*7*T-Km-*P1-bpeS*$_{K267T}$ insertion at the *glmS3* associated *att*Tn*7* site were retained.

A mini-Tn*7-P1-bpeS*$_{S29P}$ expression vector was constructed in several steps. First, pPS3189 was created as described above in the section Constructs for introduction of *bpeS*$_{P29S}$ into the chromosome. Second, a 979 bp *bpeS*$_{P29S}$ containing DNA fragment was PCR amplified utilizing Platinum Taq DNA polymerase High Fidelity and primers 2693 + 2871 and a pPS3189 template. Cloning of this fragment into pGEM-T Easy created pPS3263. Finally, a 946 bp *Ale*I-*Kpn*I fragment containing *bpeS*$_{P29S}$ was

isolated from pPS3263 and used to replace the resident *Ale*I-*Kpn*I fragment of pUC18T-mini-Tn*7*T-Km-*P1-bpeS* (or pPS3198) to form pUC18T-mini-Tn*7*T-Km-*P1- bpeS*$_{P29S}$ (pPS3269). Plasmids pPS3269 and pTNS3 were co-electroporated into Bp82.264 and Bp82.286. Strains Bp82.310 or Bp82.311 containing a PCR confirmed mini-Tn*7*T-Km-*P1-bpeS*$_{P29S}$ insertion at the *glmS3* associated *att*Tn*7* site were retained.

Whole genome sequencing

Genomic DNA from Bp82 laboratory strains and clinical isolates was isolated using the PureGene Core kit A (QIAGEN, Valencia, CA). DNA samples were prepared for paired-end sequencing on the Illumina GAIIx Genome Analyzer (Illumina, Inc, San Diego, CA) using the Kapa Biosystems library preparation kit (Woburn, MA, catalog #KK8201) protocol with an 8 bp index modification. Briefly, 2 μg double-stranded DNA from each sample was sheared to an average size of 350 bp and then input into the Kapa Illumina paired end library preparation protocol. Modified oligonucleotides (Integrated DNA Technologies, Coralville, IA) that provide 8 bp indexing capability were substituted at the appropriate step (8). Prior to sequencing the libraries were quantified with qPCR on the ABI 7900HT (Life Technologies Corporation) using the Kapa Library Quantification Kit (Catalog #KK4835). The libraries were sequenced to a read length of 100 bp on the Illumina GAIIx Genome Analyzer, yielding 13.2 M, 18.4 M and 20.8 M reads, respectively. The DNASTAR SeqMan NGen application (Madison, WI) and/or the SPANDx pipeline (9) was used to analyze the Illumina sequence data. Both tools used default settings.

References

1. **Lopez CM, Rholl DA, Trunck LA, Schweizer HP.** 2009. Versatile dual-technology system for markerless allele replacement in *Burkholderia pseudomallei*. Appl Environ Microbiol **75:**6496-6503.
2. **Choi K-H, Mima T, Casart Y, Rholl D, Kumar A, Beacham IR, Schweizer HP.** 2008. Genetic tools for select agent compliant manipulation of *Burkholderia pseudomallei*. Appl Env Microbiol **74:**1064-1075.
3. **Messing J.** 1983. New M13 vectors for cloning. MethodsEnzymol **101:**20-78.
4. **Podnecky NL, Wuthiekanun V, Peacock SJ, Schweizer HP.** 2013. The BpeEF-OprC efflux pump is responsible for widespread trimethoprim resistance in clinical and environmental *Burkholderia pseudomallei* isolates. Antimicrob Agents Chemother **57:**4381-4386.
5. **Mima T, Schweizer HP.** 2010. The BpeAB-OprB efflux pump of *Burkholderia pseudomallei* 1026b does not play a role in quorum sensing, virulence factor production, or extrusion of aminoglycosides but is a broad-spectrum drug efflux system. Antimicrob Agents Chemother **54:**3113-3120.
6. **Deshazer D, Woods DE.** 1996. Broad-host-range cloning and cassette vectors based on the R388 trimethoprim resistance gene. Biotechniques **20:**762-764.
7. **Choi K-H, Gaynor JB, White KG, Lopez C, Bosio CM, Karkhoff-Schweizer RR, Schweizer HP.** 2005. A Tn*7*-based broad-range bacterial cloning and expression system. Nat Methods **2:**443-448.
8. **Kozarewa I, Turner DJ.** 2011. 96-plex molecular barcoding for the Illumina Genome Analyzer. Methods Mol Biol **733:**279-298.
9. **Sarovich DS, Price EP.** 2014. SPANDx: a genomics pipeline for comparative analysis of large haploid whole genome re-sequencing datasets. BMC Res Notes **7:**618. DOI: 10.2147/IDR.S35529.
