# Supplementary material for: Mechanisms of Resistance to Folate Pathway Inhibitors in Burkholderia pseudomallei: Deviation from the Norm
Source: mBio. 2017 Sep 5;8(5):e01357-17. doi: 10.1128/mBio.01357-17 (PMC5587915; doi:10.1128/mBio.01357-17)
Supplement: TABLE S4 [file mbo004173469st4.pdf]

Table S4. Primers used in this study.

Primer	Sequence[a,b,c]	Source
**Cloning /deletion/sequencing**		
1726	5’-CTCTGGATCGCCTTCTCGTA	This study
1727	5’-TCAGAGCGCTTTTGAAGCTAATTCGAGATCGCGCTGAAGAACAAC	This study
1728	5’-AGGAACTTCAAGATCCCCAATTCGGCTCTCGACCACCCAGTTCT	This study
1729	5’-GTACGAGCGCCTATCTGGTC	This study
1791	5’-CGACGCATCGCGATGGAAAC	This study
1790	5’-ATGGACCGGCTGCAAGCCAT	This study
1966	5’-CTTCCGGCCTCTTTTCTTTC	(1)
1967	5’-GTGCTGATCGAGCAGATGAC	(1)
1989	5’-GGAAGTACGCGGACTTCGC	This study
1990	5’-GCATCAACCTCGGCTACACG	This study
2323	5’-CCAGATCAACGACATCATGG	This study
2324	5’-CGAGCATATAGCCCGATACC	This study
2570	5’-GGATGACTTCGGCGCTATC	This study
2571	5’-CCGTTCAACCTGACCTCAAC	This study
2572	5’-GTCTTCCGCCAGCGCTAC	This study
2573	5’-AAGCCGATTCATCTGGACAC	This study
2575	5’-CGTCGATGCGGTCTATACG	This study
2576	5’-ATCGAAGCTCGGCAGGTG	This study
2577	5’-CGCGCCTACGAGGAGTTC	This study
2592	5’-CTCGCTCACGCTGATTGC	This study
2607	5’-CGCTCGGATG*T*CGCGCGCGTCG	This study
2608	5’-CGACGCGCGCG*A*CATCCCGAGCG	This study
2618	5’-CCTGAAGCAGCAACAGCAC	This study
2619	5’-TCAGAGCGCTTTTGAAGCTAATTCGATATCGATAGCGCCGAAGTC	This study
2620	5’-GACTTCGGCGCTATCGATATCGAATTAGCTTCAAAAGCGCTCTGA	This study
2621	5’-GCTTCCTTCGCTTCGATGCGAATTGGGGATCTTGAAGTACCT	This study
2622	5’-AGGTACTTCAAGATCCCCAATTCGCATCGAAGCGAAGGAAGC	This study
2623	5’-CTTTCGCGTGAACGATCC	This study
2636	5’-AGCGAATAATCGACCGACAC	This study
2637	5’-GATGACGGACGAGGAAAGC	This study
2638	5’-CCAATATCGCGGAGGTAGAGCGGTTAGTCGCGCAGACG	This study
2639	5’-TCACTGCTTGCCGTCC**AAGCTT**AGGTGTTCGTCAGGTTGACC	This study
2640	5’-CGAGCCGCGACGAAG	This study
2641	5’-CATCGACCACGGCACG	This study
2642	5’-GGTCAACCTGACGAACACCT**AAGCTT**GGACGGCAAGCAGTGA	This study
2643	5’-CGTCTGCGCGACTAACCGCTCTACCTCCGCGATATTGG	This study
**P1 promoter expression constructs**		
1563	5’-GGGATATC**GAATTC**ACGAACCCAGTTGAC	This study
1789	5’-ATGGCTTGCAGCCGGTCCATGTCGAATCCTTCTTGTGAATC	This study
1790	5’-ATGGACCGGCTGCAAGCCAT	This study
1791	5’-CGACGCATCGCGATGGAAAC	This study
2702	5’-**AAGCTT**TTGACATAAGCCTGTTCGGTTCG	This study
2693	5’-**GGTACC**TACGCGGCCACCTGC	This study
2694	5’-GCATGGACCGCATTCAGGCAAT	This study
2696	5’-GCGGTCCATGCCATCAATCCTTCTTGTGAATC	This study
2871	5’-ATGGACCGCATTCAGGCAATGGAAGTCTTC	This study

Table S4. Continued.

Primer	Sequence	Source
**Real time PCR primers**		
Bp23S_F	5'-GTAGACCCGAAACCAGGTGA	(2)
Bp23S_R	5'-CACCCCTATCCACAGCTCAT	(2)
1524 (*bpeF)*	5'-TCCGAGTATCCGGAAGTCGT	(2)
1525 (*bpeF*)	5'-GTCCTCGACACCGTTGATCT	(2)
1814 (*bpeT*)	5'-GAGCTTTCAGGTCAACAACC	(1)
1815 (*bpeT*)	5'-GTGAGTGGAATTCGCAGAG	(1)
2779 (*bpeS*)	5'-AAGCGCTCAGGTAATCGGG	This study
2780 (*bpeS*)	5'-GGTCGAAGAGGGGATCGATTG	This study
**Tn*7* integration confirmation**		
Tn7L	5'-ATTAGCTTACGACGCTACACCC	(3)
BPGLMS1	5'-GAGGAGTGGGCGTCGATCAAC	(3)
BPGLMS2	5'-ACACGACGCAAGAGCGGAATC	(3)
BPGLMS3	5'-CGGACAGGTTCGCGCCATGC	(3)

[a]Bold indicates a newly generated restriction enzyme cleavage site.
[b]Underline indicates a homologous splicing region.
[c]Italic type indicates a nucleotide change.

References

1. **Podnecky NL, Wuthiekanun V, Peacock SJ, Schweizer HP.** 2013. The BpeEF-OprC efflux pump is responsible for widespread trimethoprim resistance in clinical and environmental *Burkholderia pseudomallei* isolates. Antimicrob Agents Chemother **57:**4381-4386.
2. **Kumar A, Mayo M, Trunck LA, Cheng AC, Currie BJ, Schweizer HP.** 2008. Expression of resistance-nodulation-cell division efflux pumps in commonly used *Burkholderia pseudomallei* strains and clinical isolates from Northern Australia. Trans Roy Soc Trop Med Hyg **102 (Suppl 1):**S145-S151.
3. **Choi K-H, Mima T, Casart Y, Rholl D, Kumar A, Beacham IR, Schweizer HP.** 2008. Genetic tools for select agent compliant manipulation of *Burkholderia pseudomallei*. Appl Env Microbiol **74:**1064-1075.
