# Supplementary material for: Mechanisms of Resistance to Folate Pathway Inhibitors in Burkholderia pseudomallei: Deviation from the Norm
Source: mBio. 2017 Sep 5;8(5):e01357-17. doi: 10.1128/mBio.01357-17 (PMC5587915; doi:10.1128/mBio.01357-17)
Supplement: TABLE S5 [file mbo004173469st5.pdf]

Table S5. Plasmids used in this study

Plasmid	Description[a]	Source
**Cloning and allele replacement vectors, and other utility plasmids**		
pGEM-T Easy	Ap^r; TA cloning vector	Promega
pCR2.1	Ap^r, Km^r; TA cloning vector	Thermofisher
pEXKm5	Km^r; allelic-exchange plasmid	(1)
pEXGm5B	Gm^r; allelic-exchange plasmid	This study
pFLPe2	Ze^r; Flp recombinase expression plasmid	(2)
pFLPe3	Km^r; Flp recombinase expression plasmid	(2)
pFKM2	Ap^r, Km^r; source of *FRT*-Km^r-*FRT* cassette	(2)
pFZE1	Zeo^r; source of *FRT-ble-FRT* cassette	(2)
p34E-Tp1	Ap^r, Tp^r; source of P1 promoter	(3)
pBADSce-T	Ze^r; I-*Sce*I homing endonuclease expression plasmid	This study
pTNS3	Ap^r; Tn*7* insertion helper plasmid	(2)
pPS1464	Ap^r, Gm^r; pUC18T-mini-Tn*7*T-Gm	(4)
pPS2280	Ap^r, Km^r; pUC18T-mini-Tn*7*T-Km	(2)
pPS2481	Ap^r, Km^r; pUC18T-mini-Tn*7*T-Km-P_{tac}	(5)
**Plasmids for efflux pump operon deletion**		
pPS2833	Km^r; pEXKm5 with Δ(*amrAB-oprA*)	This study
pPS2412	Ap^r, Km^r; pGEM-T Easy with Δ(*bpeAB-oprB*)::*FRT*-Km^r-*FRT*	(6)
pPS2899	Gm^r; pEXGm5B with Δ(*bpeAB-oprB*)	This study
pPS2591	Km^r; pEXKm5 with Δ(*bpeEF-oprC*)	(7)
**Plasmids for *bpeT* deletion**		
pPS2565	Ap^r, Km^r; pCR2.1 with *bpeT* amplified with primers 1790 + 1791	This study
pPS2567	Ap^r, Km^r;Ze^r; pCR2.1 Δ*bpeT* (*FRT-ble-FRT* between *bpeT Sal*I sites)	This study
pPS2571	Km^r; Ze^r; pEXKm5 with Δ*bpeT*::*FRT-ble-FRT* from pPS2567	This study
pPS2647	Km^r; pEXKm5 with Δ*bpeT*	This study
**Plasmids for *bpeS* deletion**		
pPS3127	Ap^r, Km^r; pGEM-T Easy with Δ*bpeS*::*FRT*-Km^r-*FRT*	This study
pPS3158	Ap^r, Gm^r; pEXGm5B with Δ*bpeS*::*FRT*-Km^r-*FRT*	This study
**Plasmids for introduction of *folA* point mutations into Bp82**		
pPS2552	Ap^r; pGEM-T Easy with $folA_{I99L}$ on 694 bp PCR fragment from Bp82.104	This study
pPS2960	Km^r; pEXKm5 with $folA_{I99L}$ containing *Eco*RI fragment from pPS2952	This study
pPS2951	Ap^r; pGEM-T Easy with $folA_{F158V}$ on 694 bp PCR fragment from Bp82.102	This study
pPS2959	Km^r; pEXKm5 with $folA_{F158V}$ containing *Eco*RI fragment from pPS2951	This study
**Plasmids for introduction of *bpeT* point mutations into Bp82**		
pPS3167	Ap^r; pGEM-T Easy with $bpeT_{L265R}$ on 2,026 bp PCR fragment from Bp82.103	This study
pPS3168	Ap^r; pGEM-T Easy with $bpeT_{C310R}$ on 2,026 bp PCR fragment from Bp82.102	This study
pPS3177	Km^r; pEXKm5 with $bpeT_{C310R}$ on 2,477 bp *Pvu*II fragment from pPS3168	This study
pPS3178	Km^r; pEXKm5 with $bpeT_{L265R}$ on 2,477 bp *Pvu*II fragment from pPS3167	This study
**Plasmids for introduction of *bpeS* point mutations into Bp82**		
pPS3097	Ap^r; pGEM-T Easy with WT *bpeS* on 1,456 bp PCR fragment from Bp82	This study
pPS3098	Ap^r; pGEM-T Easy with $bpeS_{K627T}$ on 1,456 bp PCR fragment from Bp82.104	This study
pPS3106	Apr; pPS3106 with $bpeS_{P29S}$ mutation introduced by mutagenic PCR	This study
pPS3180	Ap^r; pGEM-T Easy with WT *bpeS* region of 2,542 bp PCR fragment from Bp82	This study
pPS3186	Ap^r; pPS3180 *Eco*RV+*Xma*I *bpeS* fragment replaced with $bpeS_{P29S}$ on the same *Eco*RV+*Xma*I fragment from pPS3106	This study
pPS3187	Ap^r; pPS3180 *Eco*RV+*Xma*I *bpeS* fragment replaced with $bpeS_{K267T}$ on the same *Eco*RV+*Xma*I fragment from pPS3098	This study

Table S5 (Continued)

Plasmid	Description[a]	Source
pPS3189	Km^r; pEXKm5 with *Not*I fragment from pPS3186 containing $bpeS_{P29S}$	This study
pPS3190	Km^r; pEXKm5 with *Not*I fragment from pPS3187 containing $bpeS_{K267T}$	This study
**Plasmids for repair of $folM_{V15G}$ mutation in Bp82.202 and Bp82.204**		
pPS3099	Ap^r; pGEM-T Easy with WT *folM* on 1,560 bp PCR fragment from Bp82	This study
pPS3093	Km^r; pEXKm5 with *Not*I fragment from pPS3099 containing WT *folM*	This study
**Plasmids for repair of $bpeS_{K267T}$ mutation in Bp82.202 and Bp82.204**		
pPS3097	Ap^r; pGEM-T Easy with WT *bpeS* on 1,456 bp PCR fragment from Bp82	This study
pPS3090	Km^r; pEXKm5 with *Not*I fragment from pPS3097 containing WT *bpeS*	This study
**Plasmids for deletion of *folM***		
pPS3130	Ap^r; pGEM-T Easy with 1,236 bp Δ*folM* SOEing PCR fragment	This study
pPS3140	Ap^r, Km^r; pPS3130 with Δ*folM*::*FRT*-Km^r-*FRT* obtained by ligation of 1,514 bp *FRT*-Km^r-*FRT* fragment from pFKM2 ligated into *Hin*dIII site of SOEing product	This study
pPS3144	Gm^r, Km^r; pEXGm5B with Δ*folM*::*FRT*-Km^r-*FRT* containing *Not*I fragment from pPS3140	This study
**Plasmids for *bpeT* and *bpeS* overexpression**		
pPS2453	Ap^r; Km^r; pCR2.1 with 1,600 bp *P1-bpeT* SOEing PCR fragment	This study
pPS2463	Ap^r; pUC18T-mini-Tn*7*T-Gm-*P1-bpeT* (pPS1464 with *Nsi*I-*Nru*I fragment from pPS2453)	This study
pPS3196	Ap^r; pGEM-T Easy with *P1-bpeS* SOEing PCR fragment	This study
pPS3198	Ap^r; Km^r; pUC18T-mini-Tn*7*T-Km-*P1-bpeS* (pPS2280 with *Kpn*I+*Hin*dIII fragment from pPS3196)	This study
pPS3262	Ap^r; pGEM-T Easy with 979 bp fragment containing $bpeS_{K267T}$ amplified from pPS3190	
pPS3280	Ap^r; Km^r; pUC18T-mini-Tn*7*T-Km-*P1*-$bpeS_{K267T}$ (946 bp *Ale*I+*Kpn*I fragment of pPS3198 exchanged with the same fragment of pPS3262)	This study
pPS3263	Ap^r; pGEM-T Easy with 979 bp fragment containing $bpeS_{P29S}$ amplified from pPS3189	This study
pPS3269	Ap^r; Km^r; pUC18T-mini-Tn*7*T-Km-*P1*-$bpeS_{P29S}$ (946 bp *Ale*I+*Kpn*I fragment of pPS3198 exchanged with the same fragment of pPS3263)	This study
**Plasmids for complementation of Δ*bpeT* and Δ(*bpeEF-oprC*)**		
pPS2670	Ap^r, Km^r; pUC18T-mini-Tn*7*T-Km-P_{tac}-*bpeEF-oprC*	(7)
pPS2787	Ap^r, Km^r; pUC18T-mini-Tn*7*T-Km -P_{bpeT}-*bpeT*	(7)

[a]Ap, ampicillin; Gm, gentamicin; Km, kanamycin; Ze, Zeocin; ^{r}, resistant; WT, Bp82 wild-type sequence; *P1*, P1 integron promoter; P_{tac}, *E. coli trp-lac* operon hybrid promoter; P_{bpeT}, native *bpeT* promoter; Promega and Thermofisher are located in Madison WI and Waltham, MA, respectively.

References

1. **Lopez CM, Rholl DA, Trunck LA, Schweizer HP.** 2009. Versatile dual-technology system for markerless allele replacement in *Burkholderia pseudomallei*. Appl Environ Microbiol **75:**6496-6503.
2. **Choi K-H, Mima T, Casart Y, Rholl D, Kumar A, Beacham IR, Schweizer HP.** 2008. Genetic tools for select agent compliant manipulation of *Burkholderia pseudomallei*. Appl Env Microbiol **74:**1064-1075.

3. **Deshazer D, Woods DE.** 1996. Broad-host-range cloning and cassette vectors based on the R388 trimethoprim resistance gene. Biotechniques **20:**762-764.
4. **Choi K-H, Gaynor JB, White KG, Lopez C, Bosio CM, Karkhoff-Schweizer RR, Schweizer HP.** 2005. A Tn*7*-based broad-range bacterial cloning and expression system. Nat Methods **2:**443-448.
5. **Rholl DA, Papp-Wallace KM, Tomaras AP, Vasil ML, Bonomo RA, Schweizer HP.** 2011. Molecular investigations of PenA-mediated beta-lactam resistance in *Burkholderia pseudomallei*. Front Microbiol **2:**139. DOI:10.3389/fmicb.2011.00139.
6. **Mima T, Schweizer HP.** 2010. The BpeAB-OprB efflux pump of *Burkholderia pseudomallei* 1026b does not play a role in quorum sensing, virulence factor production, or extrusion of aminoglycosides but is a broad-spectrum drug efflux system. Antimicrob Agents Chemother **54:**3113-3120.
7. **Podnecky NL, Wuthiekanun V, Peacock SJ, Schweizer HP.** 2013. The BpeEF-OprC efflux pump is responsible for widespread trimethoprim resistance in clinical and environmental *Burkholderia pseudomallei* isolates. Antimicrob Agents Chemother **57:**4381-4386.
